# Measurement of the neutron charge radius and the role of its constituents

**DOI:** 10.1038/s41467-021-22028-z

**Published:** 2021-03-19

**Authors:** H. Atac, M. Constantinou, Z.-E. Meziani, M. Paolone, N. Sparveris

**Affiliations:** 1 Temple University, Philadelphia, PA USA; 2 Argonne National Laboratory, Lemont, IL USA; 3 New Mexico State University, Las Cruces, NM USA

**Keywords:** Experimental nuclear physics, Theoretical nuclear physics

## Abstract

The neutron is a cornerstone in our depiction of the visible universe. Despite the neutron zero-net electric charge, the asymmetric distribution of the positively- (up) and negatively-charged (down) quarks, a result of the complex quark-gluon dynamics, lead to a negative value for its squared charge radius, $\langle {r}_{{\rm{n}}}^{2}\rangle$. The precise measurement of the neutron’s charge radius thus emerges as an essential part of unraveling its structure. Here we report on a $\langle {r}_{{\rm{n}}}^{2}\rangle$ measurement, based on the extraction of the neutron electric form factor, ${G}_{{\rm{E}}}^{{\rm{n}}}$, at low four-momentum transfer squared (*Q*^2^) by exploiting the long known connection between the *N* → Δ quadrupole transitions and the neutron electric form factor. Our result, $\langle {r}_{{\rm{n}}}^{2}\rangle =-0.110\pm 0.008\,({{\rm{fm}}}^{2})$, addresses long standing unresolved discrepancies in the $\langle {r}_{{\rm{n}}}^{2}\rangle$ determination. The dynamics of the strong nuclear force can be viewed through the precise picture of the neutron’s constituent distributions that result into the non-zero $\langle {r}_{{\rm{n}}}^{2}\rangle$ value.

## Abstract (web summary)

The charge radius of nucleons provides information about their structure. Here the authors present a method, based values of neutron electric form factors, to determine the charge radius of the neutron and provide information on improving the uncertainty of neutron charge radius measurements

## Introduction

The study of the nucleon charge radius has been historically instrumental towards the understanding of the nucleon structure. In the neutron case, it is the highly complicated dynamics of the strong force between quarks and gluons, the fermionic nature of quarks and spin-orbit correlations that leads to an asymmetric distribution of u- and d-quarks in it, thus resulting in a negative value for $\langle {r}_{{\rm{n}}}^{2}\rangle$. The precise measurement of $\langle {r}_{{\rm{n}}}^{2}\rangle$ becomes a critical part of our understanding of the nucleon dynamics. Furthermore, employing new, different techniques in extracting this fundamental quantity has proven most valuable, as recently exhibited in the proton’s case: the disagreement of the proton charge radius, *r*_p_, as determined using the Lamb shift measurement in the muonic hydrogen atom^1^, with the earlier results based on the hydrogen atom and the electron scattering measurement, gave rise to the proton radius puzzle^2^. In turn, this led to a significant reassessment of the methods and analyses utilized in the proton radius extraction, and to the consideration of physics beyond the standard model as potential solutions to this discrepancy. Various atomic and nuclear physics techniques were employed for the proton *r*_p_ measurement. However, in the neutron case, the determination of $\langle {r}_{{\rm{n}}}^{2}\rangle$ is more challenging since no atomic method is possible and the electron scattering method suffers from severe limitations due to the absence of a free neutron target. Thus, the extraction of $\langle {r}_{{\rm{n}}}^{2}\rangle$ has been uniquely based on the measurement of the neutron-electron scattering length *b*_ne_, where low-energy neutrons are scattered by electrons bound in diamagnetic atoms. The $\langle {r}_{{\rm{n}}}^{2}\rangle$ measurements adopted by the particle data group (PDG)^3–6^ exhibit discrepancies, with the values ranging from $\langle {r}_{{\rm{n}}}^{2}\rangle =-0.114\pm 0.003$^4^ to $\langle {r}_{{\rm{n}}}^{2}\rangle =-0.134\pm 0.009\,({{\rm{fm}}}^{2})$^5^. Among the plausible explanations that have been suggested for this, one can find the effect of resonance corrections and of the electric polarizability, as discussed e.g., in ref. ^4^. However, these discrepancies have not been fully resolved, a direct indication of the limitations of this method.

An alternative way to determine $\langle {r}_{{\rm{n}}}^{2}\rangle$ is offered by measuring the slope of the neutron electric form factor, ${G}_{{\rm{E}}}^{{\rm{n}}}$, at *Q*^2^ → 0, which is proportional to $\langle {r}_{{\rm{n}}}^{2}\rangle$. In the past, determinations of ${G}_{{\rm{E}}}^{{\rm{n}}}$ at finite *Q*^2^ were typically carried out by measuring double polarization observables in quasi-elastic electron scattering from polarized deuterium or ^3^He targets using polarized electron beams^7–21^. However, these measurements were not able to access ${G}_{{\rm{E}}}^{{\rm{n}}}$ at a sufficiently low *Q*^2^ range so that the slope, and subsequently the $\langle {r}_{{\rm{n}}}^{2}\rangle$ can be determined.

In this work we rely on an alternative path to access ${G}_{{\rm{E}}}^{{\rm{n}}}$. It has long been known^22,23^ that the ratios of the quadrupole to the magnetic dipole transition form factors (TFFs) of the proton, *C*2/*M*1 (CMR) and *E*2/*M*1 (EMR), are related to the neutron elastic form factors ratio ${G}_{{\rm{E}}}^{{\rm{n}}}/{G}_{{\rm{M}}}^{{\rm{n}}}$. Here, we follow that path and we access ${G}_{{\rm{E}}}^{{\rm{n}}}$ at low momentum transfers from high precision measurements of the two quadrupole TFFs. The main steps of this work are summarized here for clarity. First, we extract ${G}_{{\rm{E}}}^{{\rm{n}}}$ from the quadrupole TFF data, at low momentum transfers^24–28^, utilizing the form factor relations^22,23^ determined within the SU(6) and the large-*N*_c_ frameworks. The variance of the ${G}_{{\rm{E}}}^{{\rm{n}}}$ results from the two analyses is treated as a theoretical uncertainty. The ${G}_{{\rm{E}}}^{{\rm{n}}}({Q}^{2})$ form factor is then parametrized and fitted to the data, and $\langle {r}_{{\rm{n}}}^{2}\rangle$ is determined from the ${G}_{{\rm{E}}}^{{\rm{n}}}$-slope at *Q*^2^ = 0. Finally, we perform the flavor decomposition of the neutron and the proton form factors measurements and derive the flavor dependent quark densities in the nucleon, which reveal with high precision the role of the quark contributions to the neutron charge radius.

## Results

A consequence of the SU(6) spin and flavor symmetry group that relates the nucleon and the Δ resonance leads to the following expression^22^

1 $$\frac{{G}_{{\rm{E}}}^{{\rm{n}}}({Q}^{2})}{{G}_{{\rm{M}}}^{{\rm{n}}}({Q}^{2})}=\frac{Q}{| {\bf{q}}| }\frac{2Q}{{M}_{{\rm{N}}}}\frac{1}{{n}_{{\rm{b}}}({Q}^{2})}\frac{C2}{M1}({Q}^{2})$$

where ∣**q**∣ is the virtual photon three-momentum transfer magnitude in the *γ*N center of mass frame and *M*_N_ is the nucleon mass. The *n*_b_ parametrizes the contribution from three-quark current terms, that tend to slightly increase the *C*2/*M*1 ratio (or correspondingly decrease the ${G}_{{\rm{E}}}^{{\rm{n}}}/{G}_{{\rm{M}}}^{{\rm{n}}}$), an SU(6) symmetry breaking correction that has been theoretically quantified to ~ 10%^22^ (i.e., *n*_b_ ~ 1.1). If one chooses to follow the most conservative path, a theoretical uncertainty can be assigned to this term that is equal to the full magnitude of the symmetry breaking contributions i.e., *n*_b_ = 1.1 ± 0.1. Considering the confidence with which the underlying theory is able to determine the level of the symmetry breaking contributions, the above assumption leads to a safe estimation, and most likely to an overestimation, of the theoretical uncertainty.

**Fig. 1:**
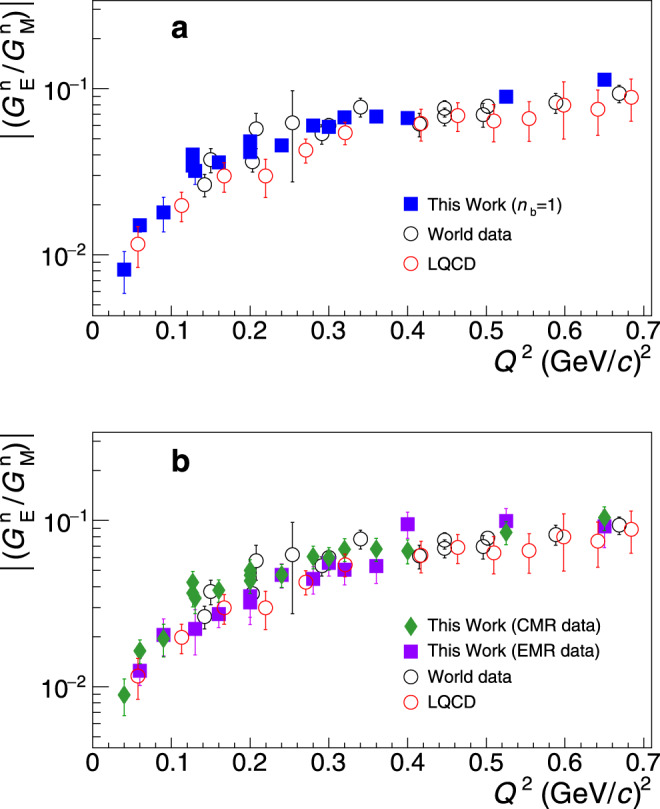
The elastic neutron form factor ratio. **a** The neutron electric to magnetic form factor ratio ${G}_{{\rm{E}}}^{{\rm{n}}}/{G}_{{\rm{M}}}^{{\rm{n}}}$: world data^7–21^ (black open circles), ratios calculated from the *N* → Δ measurements^24–32^ through Eq. (1) for *n*_b_ = 1 (filled-squares), and lattice Quantum Chromodynamics (LQCD) results (red open circles)^33^. **b** The ${G}_{{\rm{E}}}^{{\rm{n}}}/{G}_{{\rm{M}}}^{{\rm{n}}}$ results from the large-*N*_c_ analysis of the Coulomb quadrupole measurements (CMR, filled diamonds) and of the Electric quadrupole measurements (EMR, filled boxes) from the experiments^24–32^. The neutron world data (black open circles) and the LQCD results (red open circles)^33^ are the same as in panel (**a**). The error bars correspond to the total uncertainty, at the 1*σ* or 68% confidence level.

**Fig. 2:**
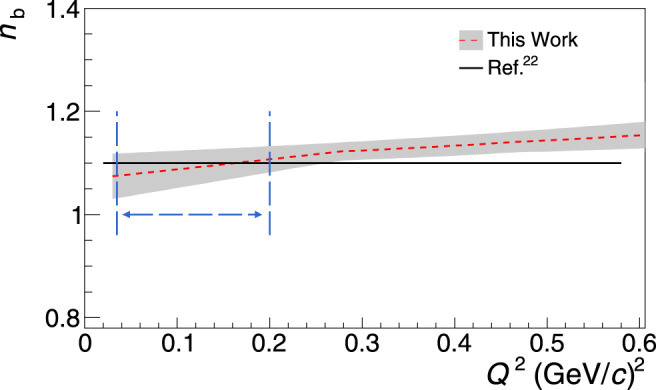
The experimentally determined symmetry breaking contributions. The breaking corrections *n*_b_ (dashed line) and the associated uncertainty *δn*_b_ (shaded band) at the 1*σ* or 68% confidence level. The solid line indicates the *n*_b_ as theoretically determined in^22^. The horizontal double-arrow marks the *Q*^2^-range where the corrections have been employed for the measurement of ${G}_{{\rm{E}}}^{{\rm{n}}}$ in this work.

In one of the first steps of this work we check the validity of the underlying theory using experimental measurements. The wealth of the TFF^24–32^ and of the ${G}_{{\rm{E}}}^{{\rm{n}}}/{G}_{{\rm{M}}}^{{\rm{n}}}$^7–21^ world data allow to quantify the magnitude of the symmetry breaking corrections from the analysis of the experimental measurements. In Fig. 1a we show the neutron ${G}_{{\rm{E}}}^{{\rm{n}}}/{G}_{{\rm{M}}}^{{\rm{n}}}$ world data^7–14,16–21^ (open black cirles), and we compare it to the ${G}_{{\rm{E}}}^{{\rm{n}}}/{G}_{{\rm{M}}}^{{\rm{n}}}$ ratios that we have derived from the TFFs *C*2/*M*1 measurements^24–32^ (filled boxes) utilizing Eq. (1) with *n*_b_ = 1 (i.e., uncorrected for the symmetry breaking contributions). By parametrizing the two data sets and then forming their ratio we can experimentally determine the magnitude of the *n*_b_(*Q*^2^) contribution. A variety of functional forms have been explored to identify the functions that can provide a good fit to the data. All the appropriate functions that offer a good fit have been considered in the determination of *n*_b_ and the variance of the results arising from the choice of the functional form is adopted as an uncertainty. The procedure is further refined using lattice Quantum Chromodynamics (LQCD) results at low momentum transfers, where neutron data do not exist. In particular, we extracted the ratio ${G}_{{\rm{E}}}^{{\rm{n}}}/{G}_{{\rm{M}}}^{{\rm{n}}}$ from numerical simulations within LQCD using the ${G}_{{\rm{E}}}^{{\rm{n}}}$ and ${G}_{{\rm{M}}}^{{\rm{n}}}$ data of ref. ^33^. The LQCD data provide further guidance on the *Q*^2^-dependence of the ${G}_{{\rm{E}}}^{{\rm{n}}}/{G}_{{\rm{M}}}^{{\rm{n}}}$ ratio based on ab-initio QCD calculations, in a region where neutron form factor data are not available. The LQCD input results to a rather small refinement of ≤0.003 in the determination of *n*_b_. The details on the determination of *n*_b_(*Q*^2^) are given in Section 2.1 of the Supplementary Information. The experimentally derived *n*_b_(*Q*^2^) is found in excellent agreement with the theoretical prediction^22^ as seen in Fig. 2; this in-turn offers further credence to the theoretical effort in ref. ^22^. Furthermore, the fitted parametrizations allow to constrain the *n*_b_(*Q*^2^) uncertainty by a factor of two compared to the most conservative *n*_b_ = 1.1 ± 0.1 (i.e., as indicated by the width of the uncertainty band in Fig. 2), but also to determine these contributions accurately at very low momentum transfers where the analysis of the current TFF data takes place for the ${G}_{{\rm{E}}}^{{\rm{n}}}$ extraction.

The LQCD results entering our analysis are compared to the experimental world data and they exhibit a very good agreement as shown in Fig. 1a. The parameters of the LQCD calculation are such that they reproduce the physical value of the pion mass. Thus, such a calculation eliminates a major source of systematic uncertainties, that is, the need of a chiral extrapolation. Furthermore, the lattice results include both the connected and disconnected diagrams, and therefore ${G}_{{\rm{E}}}^{{\rm{n}}}$ and ${G}_{{\rm{M}}}^{{\rm{n}}}$ include both valence and sea quark contributions.

**Fig. 3:**
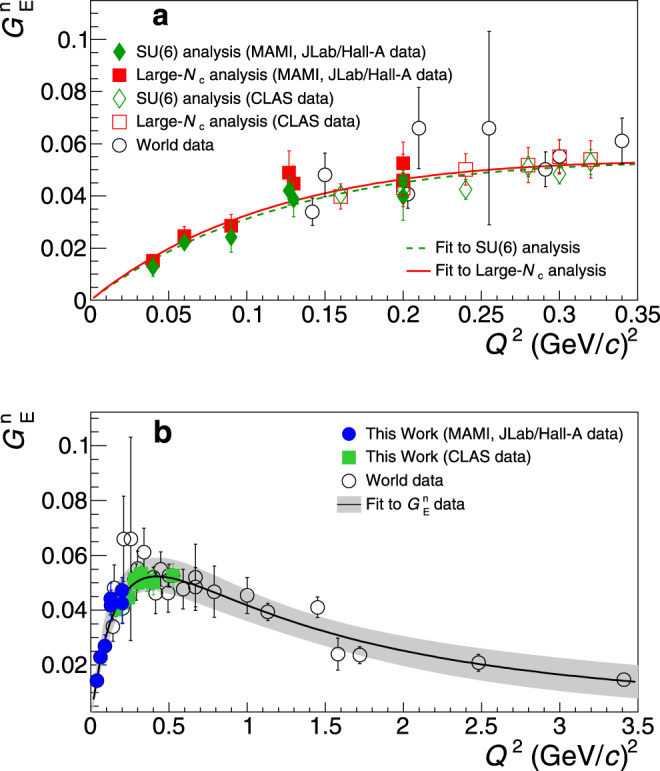
The neutron electric form factor. **a** Green diamonds: the neutron electric form factor, ${G}_{{\rm{E}}}^{{\rm{n}}}$, at low momentum transfers from the analysis based on the SU(6)^22^ and *n*_b_ determined from the world data. Red boxes: the ${G}_{{\rm{E}}}^{{\rm{n}}}$ results from the analysis based on the large-*N*_c_^23^. The fit to the data from the parametrization of Eq. (5) is shown with the dashed and the solid curves, respectively. The filled symbols (diamonds/boxes) correspond to the analysis of the data from ref. ^24–28^ (MAMI, JLab/Hall-A data) and the open ones to that of ref. ^31^ (CLAS data). **b** Blue circles: the final ${G}_{{\rm{E}}}^{{\rm{n}}}$ results at low momentum transfers, extracted from the weighted average of the SU(6) and the large-*N*_c_ analysis results. The variance of the two data sets is quantified as a theoretical uncertainty. The solid curve shows the fit to the data from the parametrization of Eq. (5), with its uncertainty (shaded band). The ${G}_{{\rm{E}}}^{{\rm{n}}}$ world data (open-circles)^7–21^ are shown. The extracted ${G}_{{\rm{E}}}^{{\rm{n}}}$ from the analysis of the CLAS measurements^31^ at intermediate momentum transfers is also shown (green boxes). The error bars correspond to the total uncertainty, at the 1*σ* or 68% confidence level.

In our analysis we have extracted the ${G}_{{\rm{E}}}^{{\rm{n}}}$ under two scenarios; in one case we consider the conservative path where *n*_b_ = 1.1 ± 0.1, while in the second we utilize the *n*_b_ as we have determined it from the experimental world data. The two sets of results come to an agreement at the ≤3% level; this is much smaller than to the overall ${G}_{{\rm{E}}}^{{\rm{n}}}$ uncertainty. A slightly improved ${G}_{{\rm{E}}}^{{\rm{n}}}$ uncertainty is obtained in the latter case due to an improved level of the *n*_b_ uncertainty, when these contributions are determined from the world data. The ${G}_{{\rm{E}}}^{{\rm{n}}}$ uncertainty of our results is driven by the following sources: (i) Experimental (statistical and systematic) uncertainties in the determination of the *C*2/*M*1 ratio. (ii) Uncertainties in the determination of *C*2/*M*1 due to the presence of non-resonant pion electro-production amplitudes that interfere with the extraction of the resonant amplitudes. These effects were studied by employing theoretical pion electro-production models in the data analysis; they were further investigated experimentally by measuring *C*2/*M*1 through an alternative reaction channel, the $p(e,e^{\prime} p)\gamma$^28^, where one employs a different theoretical framework for the ratio extraction (see Supplementary Information, Section 1). (iii) The uncertainty of the symmetry breaking terms *δn*_b_, as discussed above. (iv) The uncertainty introduced by the choice of the ${G}_{{\rm{M}}}^{{\rm{n}}}$-parametrization, in order to extract the ${G}_{{\rm{E}}}^{{\rm{n}}}$ from the ${G}_{{\rm{E}}}^{{\rm{n}}}/{G}_{{\rm{M}}}^{{\rm{n}}}$ ratio (as typically done in such cases e.g.,^9,17^). In this work we have used the one from ref. ^34^ and we have quantified the associated uncertainty by repeating the analysis with alternative parametrizations. We have found a ~0.5% effect, which is rather small compared with the total ${G}_{{\rm{E}}}^{{\rm{n}}}$ uncertainty. The ${G}_{{\rm{E}}}^{{\rm{n}}}$ results are displayed in Fig. 3a.

The relation between ${G}_{{\rm{E}}}^{{\rm{n}}}$ and the quadrupole transition form factors has also been established through large-*N*_c_ relations^23^. The relations take the form

2 $$\frac{E2}{M1}({Q}^{2})={\left(\frac{{M}_{{\rm{N}}}}{{M}_{\Delta }}\right)}^{3/2}\frac{{M}_{\Delta }^{2}-{M}_{{\rm{N}}}^{2}}{2{Q}^{2}}\frac{{G}_{{\rm{E}}}^{{\rm{n}}}({Q}^{2})}{{F}_{2}^{{\rm{p}}}({Q}^{2})-{F}_{2}^{{\rm{n}}}({Q}^{2})}$$

3 $$\frac{C2}{M1}({Q}^{2})={\left(\frac{{M}_{{\rm{N}}}}{{M}_{\Delta }}\right)}^{3/2}\frac{{Q}_{+}{Q}_{-}}{2{Q}^{2}}\frac{{G}_{{\rm{E}}}^{{\rm{n}}}({Q}^{2})}{{F}_{2}^{{\rm{p}}}({Q}^{2})-{F}_{2}^{{\rm{n}}}({Q}^{2})}$$

where ${F}_{2}^{({\rm{pn}})}$ are the nucleon Pauli form factors, *M*_Δ_ is the mass of the Δ, and ${Q}_{\pm }={({({M}_{\Delta }\pm {M}_{{\rm{N}}})}^{2}+{Q}^{2})}^{\frac{1}{2}}$. Here one is free from any additional correction terms, such as the symmetry breaking contributions of Eq. (1). Another advantage is that the experimental database is extended to include the Electric quadrupole (E2) transition, which in turn allows for an improved extraction of ${G}_{{\rm{E}}}^{{\rm{n}}}$. Being able to extract ${G}_{{\rm{E}}}^{{\rm{n}}}$ independently through the Coulomb and the Electric quadrupole transitions offers a strong experimental test to the validity of the large-*N*_c_ relations and allows to quantify their level of theoretical uncertainty. The above relations come with a 15% theoretical uncertainty^23^ that is treated accordingly in the ${G}_{{\rm{E}}}^{{\rm{n}}}$ analysis. The ${G}_{{\rm{E}}}^{{\rm{n}}}$ extraction from the Coulomb and from the Electric quadrupole transitions agree nicely within that level, as can be seen in Fig. 1b, and validate this level of uncertainty. For the well-known ${G}_{{\rm{E}}}^{{\rm{p}}}$, ${G}_{{\rm{M}}}^{{\rm{p}}}$, and ${G}_{{\rm{M}}}^{{\rm{n}}}$ that enter in the expressions through the Pauli form factors we have used recent parametrizations. For the ${G}_{{\rm{M}}}^{{\rm{p}}}$ and ${G}_{{\rm{M}}}^{{\rm{n}}}$ we used ref. ^34^. For ${G}_{{\rm{E}}}^{{\rm{p}}}$ we performed an updated parametrization so that we may include recent measurements from ref. ^35^ that were not yet available in ref. ^34^ (see Supplementary Information, Section 4). For the large-*N*_c_ analysis the final results integrate both of the quadrupole transition form factors from each experiment, when both of them were simultaneously measured, into one ${G}_{{\rm{E}}}^{{\rm{n}}}$ measurement (see Supplementary Information, Section 2.2). The extracted ${G}_{{\rm{E}}}^{{\rm{n}}}$ results from the large-*N*_c_ analysis are displayed in Fig. 3a and are compared to the results from the SU(6) analysis in the same figure. The SU(6) analysis is in agreement with the large-*N*_c_ analysis ${G}_{{\rm{E}}}^{{\rm{n}}}$ results. For our final ${G}_{{\rm{E}}}^{{\rm{n}}}$ result we consider the weighted average of the two values, as shown in Fig. 3b. The variance of the two values is treated as an additional ${G}_{{\rm{E}}}^{{\rm{n}}}$ theoretical uncertainty, and is accounted for accordingly in the $\langle {r}_{{\rm{n}}}^{2}\rangle$ extraction.

The neutron mean square charge radius is related to the slope of the neutron electric form factor as *Q*^2^ → 0 through

4 $$\langle {r}_{{\rm{n}}}^{2}\rangle ={\left.-6\frac{d{G}_{{\rm{E}}}^{{\rm{n}}}({Q}^{2})}{d{Q}^{2}}\right|}_{{Q}^{2}\to 0}.$$

**Fig. 4:**
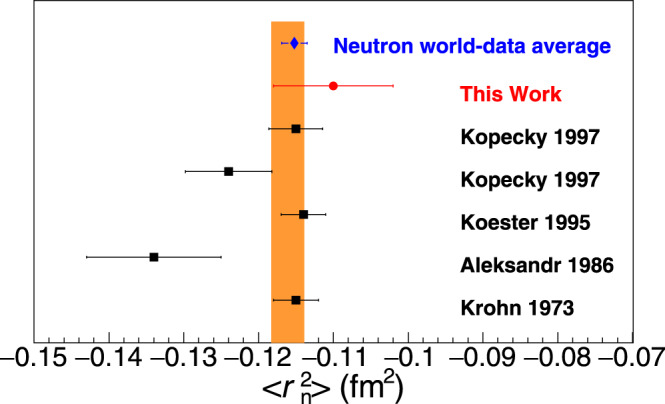
The neutron mean square charge radius. The $\langle {r}_{{\rm{n}}}^{2}\rangle$ measurement from this work (red circle), with the error bar corresponding to the total uncertainty at the 1*σ* or 68% confidence level, and from references^3–6^ (black box) included in the PDG analysis for $\langle {r}_{{\rm{n}}}^{2}\rangle$. The orange-band indicates the PDG averaged $\langle {r}_{{\rm{n}}}^{2}\rangle$ value. The new weighted average of the world data is also shown (blue diamond) when the new $\langle {r}_{{\rm{n}}}^{2}\rangle$ measurement reported in this work is included in the calculation.

In order to determine the charge radius the data have to be fitted to a functional form, and the slope has to be determined at *Q*^2^ = 0. It is important that a proper functional form is identified so that model dependent biases to the fit are avoided. In the past, the experimental data would allow to explore functional forms for ${G}_{{\rm{E}}}^{{\rm{n}}}({Q}^{2})$ with only two free parameters, that were lacking the ability to determine the neutron charge radius. The updated data allow to introduce an additional free parameter and to extract the $\langle {r}_{{\rm{n}}}^{2}\rangle$ from measurements of the ${G}_{{\rm{E}}}^{{\rm{n}}}$ that was not possible previously. Our studies have shown that

5 $${G}_{{\rm{E}}}^{{\rm{n}}}({Q}^{2})={(1+{Q}^{2}/A)}^{-2}\frac{B\tau }{1+C\tau },$$

is the most robust function for the radius extraction, where $\tau ={Q}^{2}/4{M}_{{\rm{N}}}^{2}$, and *A*, *B*, *C* are free parameters (see Supplementary Information, Section 3). Our fits employ the ${G}_{{\rm{E}}}^{{\rm{n}}}$ data discussed in this work as well as the ${G}_{{\rm{E}}}^{{\rm{n}}}$ world data from^7–21^. The function describes the data very well, with a reduced *χ*^2^ of 0.74. The parameters obtained are *A* = 0.505 ± 0.079 (GeV/*c*)^2^, *B* = 1.655 ± 0.126, *C* = 0.909 ± 0.583, and *Q*^2^ in units of (GeV/*c*)^2^, leading to a value of $\langle {r}_{{\rm{n}}}^{2}\rangle =-0.110\pm 0.008\,({{\rm{fm}}}^{2})$, as shown in Fig. 4. When the uncertainty of the symmetry breaking contributions in the SU(6) analysis is treated conservatively (i.e., *n*_b_ = 1.1 ± 0.1) the final result becomes $\langle {r}_{{\rm{n}}}^{2}\rangle =-0.109\pm 0.009\,({{\rm{fm}}}^{2})$ with a reduced *χ*^2^ of 0.74. Here we observe that the $\langle {r}_{{\rm{n}}}^{2}\rangle$-uncertainty is not affected significantly by the different treatment of the symmetry breaking contributions in the two cases.

The charge radius extraction is further explored through fits that are constrained within a limited range at low *Q*^2^ where ${G}_{{\rm{E}}}^{{\rm{n}}}$ remains monotonic, namely from *Q*^2^ = 0 to 0.4 (GeV/*c*)^2^. In the fits to the data, the functional forms can be divided into two groups, those based on polynomials with varying orders and those that are based on rational forms (see Supplementary Information, Section 3.1). For the charge radius, the weighted average is extracted separately for each one of the two groups. A systematic uncertainty is also quantified within each group (i.e., a model uncertainty of the group) from the weighted variance of the results from all the fits within the group. The results from the two groups tend to have a similar overall uncertainty. A small systematic difference of the two group’s central $\langle {r}_{{\rm{n}}}^{2}\rangle$ values is observed, as studies over a varying fitting range have shown. For that reason a third uncertainty is determined: here we consider the spread of the two central values as indicative of the uncertainty that is associated with the choice of the group. Therefore, the final result is given by the average of the two group values for $\langle {r}_{{\rm{n}}}^{2}\rangle$, while the half of the difference of the two values is assigned as an additional uncertainty. The details of the studies are presented in the Supplementary Information, Sections 3.1 and 3.2. The results from the low-*Q*^2^ fits for all groups of functions and for variations to the fitting range are shown in the Supplementary Information, Table 7 and Table 8. The low-*Q*^2^ fits confirm the $\langle {r}_{{\rm{n}}}^{2}\rangle$ extraction from the fits over the complete ${G}_{{\rm{E}}}^{{\rm{n}}}$ database, but they are vulnerable to model uncertainties that are associated with the choice of the fitted parametrization and they are not able to improve the $\langle {r}_{{\rm{n}}}^{2}\rangle$ extraction. The studies indicate that more ${G}_{{\rm{E}}}^{{\rm{n}}}$ measurements are needed at lower momentum transfers so that a more competitive extraction can become possible from the low-*Q*^2^ fits. This comes as no surprise when one considers the corresponding case for the proton, in which case the charge radius extraction from fits within a limited *Q*^2^-range required measurements at significantly smaller momentum transfers, namely at *Q*^2^ = 0.0002 (GeV/*c*)^2^  −  0.06 (GeV/*c*)^2^ ^35^.

The quadrupole TFF data at low momentum transfers^24–28^, acquired at MAMI and at JLab/Hall-A, provide the critical ${G}_{{\rm{E}}}^{{\rm{n}}}$ data that were missing lower than *Q*^2^ = 0.20 (GeV/*c*)^2^ and make the $\langle {r}_{{\rm{n}}}^{2}\rangle$ extraction possible. We now explore the potential of extending the current analysis to higher momentum transfers. This study aims to observe the effect of the $\langle {r}_{{\rm{n}}}^{2}\rangle$ extraction if we enrich the ${G}_{{\rm{E}}}^{{\rm{n}}}$ database with measurements in the region where ${G}_{{\rm{E}}}^{{\rm{n}}}$ data already exist, higher than *Q*^2^ = 0.20 (GeV/*c*)^2^. The relations between the ${G}_{{\rm{E}}}^{{\rm{n}}}$ and the quadrupole transition form factors hold on very solid ground in the low *Q*^2^ region, i.e., lower than *Q*^2^ = 0.20 (GeV/*c*)^2^. On the other hand, they tend to hold less well at high momentum transfers. The relations do not come with a sharp *Q*^2^ cut–off-value after which they do not hold, but one should avoid the *Q*^2^ = 1 (GeV/*c*)^2^ region where larger theoretical uncertainties could bias the charge radius extraction. In extending the database higher in *Q*^2^, care has to be given so that any additional data at intermediate momentum transfers will benefit the fits without compromising the $\langle {r}_{{\rm{n}}}^{2}\rangle$ extraction by the gradually increasing theoretical uncertainties. Our studies showed that when we integrate in the analysis the ${G}_{{\rm{E}}}^{{\rm{n}}}$ data that we extract from the CLAS measurements up to *Q*^2^ = 0.52 (GeV/*c*)^2^ ^31^ (see Fig. 3b, green boxes) we find that $\langle {r}_{{\rm{n}}}^{2}\rangle =-0.107\pm 0.007\,({{\rm{fm}}}^{2})$, compared with $\langle {r}_{{\rm{n}}}^{2}\rangle =-0.110\pm 0.008\,({{\rm{fm}}}^{2})$ when these additional data are not included (see the Supplementary Information, Section 3.2 for details). Last, when the same data-set is included in the fits within the limited low-*Q*^2^ range, as discussed in the previous paragraph, we find that $\langle {r}_{{\rm{n}}}^{2}\rangle =-0.111\pm 0.006\pm 0.00{2}_{{\rm{mod}}}\pm 0.00{4}_{{\rm{group}}}\,({{\rm{fm}}}^{2})$. Here the last two uncertainties (mod and group) are model-related uncertainties associated with the choice of the fitted parametrization (see Supplementary Information, Section 3.2 for details). We do not observe any additional benefit by extending the measurements higher in *Q*^2^ since the fits’ uncertainties do not improve when more data are included up to *Q*^2^ = 1 (GeV/*c*)^2^. In conclusion, a small benefit to the charge radius uncertainty can be observed when additional data up to *Q*^2^ = 0.5 (GeV/*c*)^2^ are utilized for the charge radius extraction. If one decides to eliminate any risk of introducing theoretical bias from the inclusion of the intermediate momentum transfer measurements for the final result, one can conservatively adopt the analysis that does not include these additional data, namely $\langle {r}_{{\rm{n}}}^{2}\rangle =-0.110\pm 0.008\,({{\rm{fm}}}^{2})$.

## Discussion

Our analysis and results offers valuable input towards addressing long standing unresolved $\langle {r}_{{\rm{n}}}^{2}\rangle$ discrepancies of the *b*_ne_-measurements, which display a ≈10% tension between the results, suggesting that there are still unidentified systematic uncertainties associated with this method of extraction. Our measurement is in disagreement with ref. ^5^ and supports the results of refs. ^3,4^. Considering that here we cross check $\langle {r}_{{\rm{n}}}^{2}\rangle$ using a different extraction method, there is a strong argument so as to exclude the value of ref. ^5^ from the world data average. In such a case, the new weighted average value of the world data when we include our measurement and we exclude the one of ref. ^5^, becomes $\langle {r}_{{\rm{n}}}^{2}\rangle =-0.1152\pm 0.0017\,({{\rm{fm}}}^{2})$. Based on the current work, the particle data book value of $\langle {r}_{{\rm{n}}}^{2}\rangle =-0.1161\pm 0.0022\,({{\rm{fm}}}^{2})$ is adjusted by ~1% and improves its uncertainty by ~23%. We also note that our result agrees very well with a recent $\langle {r}_{{\rm{n}}}^{2}\rangle$ calculation that is based on the determination of the deuteron structure radius in chiral effective field theory and utilizes atomic data for the difference of the deuteron and proton charge radii^36^.

**Fig. 5:**
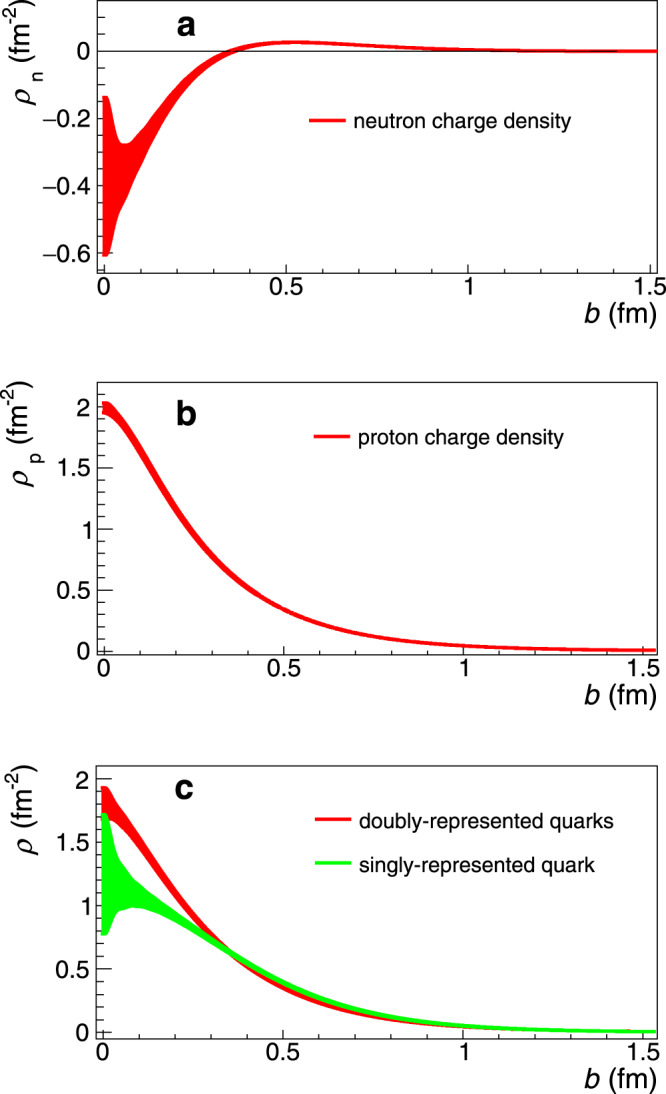
The nucleon charge densities. **a** The neutron charge density *ρ*_n_. **b** The proton charge density *ρ*_p_. **c** Flavor decomposition of the nucleon charge densities: the doubly-represented (red) and singly-represented (green) charge densities in the nucleon. Each is normalized to unity.

The neutron’s non-zero mean charge radius is a direct consequence of the asymmetric distribution of the positively-charged (up) and of the negatively-charged (down) quarks in the system, a consequence of the non-trivial quark gluon dynamics of the strong force. The quark distributions offer a detailed view as to how the non-zero $\langle {r}_{{\rm{n}}}^{2}\rangle$ value arises. Here, one has to work on the infinite-momentum frame^37^ since it offers the inherent advantage that a true transverse charge density can be properly defined as the matrix element of a density operator between identical initial and final states. We find that the results of our analysis on $\langle {r}_{{\rm{n}}}^{2}\rangle$ are particularly sensitive to the neutron’s long-distance structure, and offer a significant improvement (factor of 2) in the precision of the neutron charge density at its surface (see Supplementary Information Fig. 9). We extract the neutron and the proton charge densities at the infinite-momentum-frame from the most recent nucleon form factor parametrizations, where for ${G}_{{\rm{E}}}^{{\rm{n}}}$ we use the one determined in this work. The details are presented in the Supplementary Information, Section 4. The extracted neutron and proton charge densities are shown in Fig. 5. From the two nucleon densities, invoking charge symmetry, and neglecting the $s\bar{s}$ contribution, we derive the *u*- and *d*-quark densities with an improved precision as shown in Fig. 5 (see Supplementary Information, Section 4 for details). The flavor dependent densities show that the singly-represented quark in the nucleon has a wider distribution compared with the doubly-represented quarks, which in turn exhibit a larger central quark density. Although the concentration of the two negatively charged quarks at the center of the neutron may appear to contradict the negative sign of the neutron’s mean square charge radius, this is not truly the case. The 3D Breit frame and 2D infinite-momentum distributions are directly related to each other and the apparent discrepancies between the distributions in the two frames simply result from kinematical artifacts associated with spin^38^. The effect is rather dramatic in the neutron, where the rest-frame magnetization is large and negative. The contribution it induces competes with the convection contribution and gradually changes the sign at the center of the charge distribution as one increases the momentum of the neutron. Thus, the appearance of a negative region around the center of the neutron charge distribution in the infinite-momentum frame is just a manifestation of the contribution induced by the rest-frame magnetization.

In conclusion, we report on an alternative measurement of the neutron charge radius, based on the measurement of the neutron electric form factor ${G}_{{\rm{E}}}^{{\rm{n}}}$. An alternative path to the measurements based on the scattering of neutrons by electrons bound in diamagnetic atoms is presented. Our value of $\langle {r}_{{\rm{n}}}^{2}\rangle =-0.110\pm 0.008\,({{\rm{fm}}}^{2})$ offers valuable input towards addressing long standing unresolved discrepancies in the $\langle {r}_{{\rm{n}}}^{2}\rangle$ measurements, rejects earlier measurements, and improves the precision of the $\langle {r}_{{\rm{n}}}^{2}\rangle$ world data average value. Furthermore, our data offer access to the associated dynamics of the strong nuclear force through the precise mapping of the quark distributions in the neutron that contribute to its non-zero charge radius. The current work lays the path for $\langle {r}_{{\rm{n}}}^{2}\rangle$ measurements of higher precision. New experimental proposals based on this method, e.g., Jefferson Lab LOI 12-20-002, offer to improve the precision of the $\langle {r}_{{\rm{n}}}^{2}\rangle$ measurement by nearly a factor of 2. Future experimental efforts will be able to utilize upgraded experimental setups that will fully exploit the advantages of this method. In particular, pushing the low momentum transfer limits of high precision measurements can lead to a further improvement in the precision of the $\langle {r}_{{\rm{n}}}^{2}\rangle$ extraction.

## Methods

Here we extract ${G}_{{\rm{E}}}^{{\rm{n}}}$, at low momentum transfers, from measurements of the quadrupole transition form factors. The neutron charge radius is then extracted from the slope of ${G}_{{\rm{E}}}^{{\rm{n}}}$ at *Q*^2^ = 0. Utilizing the ${G}_{{\rm{E}}}^{{\rm{n}}}$ data and the world data for the nucleon elastic form factors the flavor decomposition of the nucleon electromagnetic form factors is performed, and the u- and d-quark distributions in the nucleon are extracted. The main steps of this work are as follows:

1. We extract ${G}_{{\rm{E}}}^{{\rm{n}}}$ from the Coulomb quadrupole and the Electric quadrupole transition form factor data at low momentum transfers^24–27^ utilizing the form factor relations (Eqs. (1–3)) that have been determined within the SU(6)^22^ and the large-*N*_c_^23^ frameworks. The symmetry breaking corrections, *n*_b_(*Q*^2^), in Eq. (1) are determined experimentally from the world data (elastic neutron form factors and *N* → Δ transition form factors) and are further refined using state of the art Lattice QCD calculations. Parametrizations are used for the well known ${G}_{{\rm{E}}}^{{\rm{p}}}$, ${G}_{{\rm{M}}}^{{\rm{p}}}$, and ${G}_{{\rm{M}}}^{{\rm{n}}}$ form factors. Multiple parametrizations are employed so as to quantify the corresponding uncertainty introduced into the ${G}_{{\rm{E}}}^{{\rm{n}}}$ extraction.
2. The final ${G}_{{\rm{E}}}^{{\rm{n}}}$ values are extracted by the weighted average of the SU(6)^22^ and the large-*N*_c_^23^ analysis results. The variance between the results of the two methods is treated as a theoretical uncertainty.
3. The neutron mean square charge radius $\langle {r}_{{\rm{n}}}^{2}\rangle$ is obtained from Eq. (4) by fitting the ${G}_{{\rm{E}}}^{{\rm{n}}}$ data to the functional form in Eq. (5) and determining the slope at *Q*^2^ = 0. This functional form was shown to be the most robust function for the radius extraction from the neutron data. The fit employs additional ${G}_{{\rm{E}}}^{{\rm{n}}}$ data reported here and the ${G}_{{\rm{E}}}^{{\rm{n}}}$ world data extending to higher momentum transfers.
4. The neutron and proton densities are derived at the infinite momentum frame through
  6 $$\rho (b)=\int_{0}^{\infty }\frac{dQQ}{2\pi }{J}_{0}(Qb)\frac{{G}_{{\rm{E}}}({Q}^{2})+\tau {G}_{{\rm{M}}}({Q}^{2})}{1+\tau }$$
  where *b* is the transverse distance, *τ* = *Q*^2^/4*m*^2^ and *J*_0_ the 0^th^ order cylindrical Bessel function. Here we utilize the most recent parametrizations for the nucleon form factors, where for ${G}_{{\rm{E}}}^{{\rm{n}}}$ we use the one derived in this work. From the neutron and proton densities, invoking charge symmetry, and neglecting the $s\bar{s}$ contribution, we then extract the *u*- and *d*-quark densities in the proton (or doubly-represented and singly-represented quarks in the nucleon, respectively), where
  7 $${\rho }_{{\rm{u}}}(b)={\rho }_{{\rm{p}}}(b)+{\rho }_{{\rm{n}}}(b)/2$$
  and
  8 $${\rho }_{{\rm{d}}}(b)={\rho }_{{\rm{p}}}(b)+2{\rho }_{{\rm{n}}}(b).$$

## Supplementary information

Supplementary Information

## Data Availability

All the relevant data in this work are available from the authors upon request. The data for the quadrupole TFFs used in this work are publicly available in their original publications^24–28,31^ where they are described in detail. The raw data from these experiments are archived in Jefferson Laboratory’s mass storage silo and at Temple University, Department of Physics. The ${G}_{{\rm{E}}}^{{\rm{n}}}$ data that we have used for the $\langle {r}_{{\rm{n}}}^{2}\rangle$ extraction are available in the following sources: (i) The new ${G}_{{\rm{E}}}^{{\rm{n}}}$ data that we have derived in this work, from the analysis of the quadrupole TFFs, are available in the Supplementary Information document. (ii) The previously published ${G}_{{\rm{E}}}^{{\rm{n}}}$ world-data are available in their original publications^7–21^.

## Code availability

The data analysis uses the standard C++ ROOT framework, which was developed at CERN and is freely available at https://root.cern.ch. The computer codes used for the data analysis and for the generation of plots are available upon request.

## References

[1] Pohl R (2010). The size of the proton. Nature.

[2] Pohl R, M GA, Gilman R, Pachucki K (2013). Muonic hydrogen and the proton radius puzzle. Ann. Rev. Nucl. Part. Sci..

[3] Kopecky S (1997). Neutron charge radius determined from the energy dependence of the neutron transmission of liquid Pb-208 and Bi-209. Phys. Rev..

[4] Koester L (1995). Neutron electron scattering length and electric polarizability of the neutron derived from cross-sections of bismuth and of lead and its isotopes. Phys. Rev..

[5] Aleksandrov YuA, Vrana M, Manrique GJ, Machekhina TA, Sedlakova LN (1986). Neutron rms radius and electric polarizability from data on the interaction of slow neutrons with bismuth. Sov. J. Nucl. Phys..

[6] Krohn VE, Ringo GR (1973). Reconsiderations of the electron - neutron scattering length as measured by the scattering of thermal neutrons by noble gases. Phys. Rev..

[7] Madey R (2003). Measurements of $${G}_{E}^{{\rm{n}}}/{G}_{M}^{{\rm{n}}}$$ from the $${\,}^{2}H{(\overrightarrow{e},{e}^{\prime},\overrightarrow{n})}^{1}H$$ reaction to *Q*^2^ = 1.45 (GeV/*c*)^2^. Phys. Rev. Lett..

[8] Schlimme BS (2013). Measurement of the neutron electric to magnetic form factor ratio at *Q*^2^ = 1.58 GeV^2^ using the reaction $${\,}^{3}\overrightarrow{He}(\overrightarrow{e},e^{\prime} n)pp$$. Phys. Rev. Lett..

[9] Riordan S (2010). Measurements of the Electric Form Factor of the Neutron up to *Q*^2^ = 3.4 GeV^2^ using the Reaction $${\,}^{3}\overrightarrow{He}(\overrightarrow{e},e^{\prime} n)pp$$. Phys. Rev. Lett..

[10] Glazier DI (2005). Measurement of the electric form-factor of the neutron at *Q*^2^ = 0.3 (GeV/*c*)^2^ to 0.8 (GeV/*c*)^2^. Eur. Phys. J..

[11] Plaster B (2006). Measurements of the neutron electric to magnetic form-factor ratio *G*_*E**n*_/*G*_*M**n*_ via the $${\,}^{2}H{(\overrightarrow{e},{e}^{\prime},\overrightarrow{n})}^{1}H$$ reaction to *Q*^2^ = 1.45 (GeV/*c*)^2^. Phys. Rev..

[12] Zhu H (2001). A measurement of the electric form-factor of the neutron through $$\overrightarrow{d}(\overrightarrow{e},{e}^{\prime}n)p$$ at *Q*^2^ = 0.5 (GeV/*c*)^2^. Phys. Rev. Lett..

[13] Warren G (2004). Measurement of the electric form-factor of the neutron at *Q*^2^ = 0.5 and 1.0 GeV^2^/*c*^2^. Phys. Rev. Lett..

[14] Rohe D (1999). Measurement of the neutron electric form-factor *G*_(*e**n*)_ at 0.67(GeV/*c*)^2^ via $${\,}^{3}\overrightarrow{He}(\overrightarrow{e},e^{\prime} n)$$. Phys. Rev. Lett..

[15] Passchier I (2000). The Charge form-factor of the neutron from the reaction polarized $${\,}^{2}H(\overrightarrow{e},e,{e}^{\prime}n)p$$. Nucl. Phys. A.

[16] Bermuth J (2003). The Neutron charge form-factor and target analyzing powers from $${\,}^{3}\overrightarrow{He}(\overrightarrow{e},{e}^{\prime}n)$$ scattering. Phys. Lett..

[17] Geis E (2008). The Charge form factor of the neutron at low momentum transfer from the $$2\overrightarrow{H}(\overrightarrow{e},{e}^{\prime}n)p$$ reaction. Phys. Rev. Lett..

[18] Eden T (1994). Electric form factor of the neutron from the $${\,}^{2}H{(\overrightarrow{e},{e}^{\prime},\overrightarrow{n})}^{1}H$$ reaction at *Q*^2^ = 0.255 (GeV/*c*)^2^. Phys. Rev. C.

[19] Ostrick M (1999). Measurement of the neutron electric form factor *G*_*E*,*n*_ in the Quasifree $${\,}^{2}H(\overrightarrow{{e}},{{e}}^{\prime}\overrightarrow{{n}}){p}$$ Reaction. Phys. Rev. Lett..

[20] Golak J, Ziemer G, Kamada H, Witała H, Glöckle W (2001). Extraction of electromagnetic neutron form factors through inclusive and exclusive polarized electron scattering on a polarized ^3^He target. Phys. Rev. C.

[21] Herberg C (1999). Determination of the neutron electric form-factor in the D(e,e’ n)p reaction and the influence of nuclear binding. Eur. Phys. J..

[22] Buchmann AJ (2004). Electromagnetic N —> Δ transition and neutron form-factors. Phys. Rev. Lett..

[23] Pascalutsa V, Vanderhaeghen M (2007). Large-*N*_c_ relations for the electromagnetic nucleon-to-Δ form factors. Phys. Rev..

[24] Blomberg A (2016). Electroexcitation of the Δ^+^(1232) at low momentum transfer. Phys. Lett..

[25] Stave S (2006). Lowest *Q*^2^ Measurement of the *γ** p —> Δ Reaction: probing the pionic contribution. Eur. Phys. J..

[26] Sparveris N (2013). Measurements of the *γ**p → Δ reaction at low *Q*^2^. Eur. Phys. J..

[27] Sparveris NF (2007). Determination of quadrupole strengths in the *γ***p* → Δ(1232) transition at *Q*^2^ = 0.20 (GeV/*c*)^2^. Phys. Lett..

[28] Blomberg A (2019). Virtual Compton Scattering measurements in the nucleon resonance region. Eur. Phys. J. A.

[29] Sparveris NF (2005). Investigation of the conjectured nucleon deformation at low momentum transfer. Phys. Rev. Lett..

[30] Elsner D (2006). Measurement of the LT-asymmetry in *π*^0^ electroproduction at the energy of the Δ(1232) resonance. Eur. Phys. J..

[31] Aznauryan IG (2009). Electroexcitation of nucleon resonances from CLAS data on single pion electroproduction. Phys. Rev..

[32] Kelly JJ (2007). Recoil polarization measurements for neutral pion electroproduction at *Q*^2^ = 1 (GeV/*c*)^2^ near the Delta resonance. Phys. Rev..

[33] Alexandrou C (2019). Proton and neutron electromagnetic form factors from lattice QCD. Phys. Rev..

[34] Ye Z, Arrington J, Hill RJ, Lee G (2018). Proton and neutron electromagnetic form factors and uncertainties. Phys. Lett..

[35] Xiong W (2019). A small proton charge radius from an electron-proton scattering experiment. Nature.

[36] Filin AA (2020). Extraction of the neutron charge radius from a precision calculation of the deuteron structure radius. Phys. Rev. Lett..

[37] Miller GA (2007). Charge density of the neutron and proton. Phys. Rev. Lett..

[38] Lorce, C. Charge Distributions of Moving Nucleons. *Phys. Rev. Lett*. 125(23), 232002 (2020). 10.1103/PhysRevLett.125.232002 33337172

