# Author Correction: Measurement of the neutron charge radius and the role of its constituents

**DOI:** 10.1038/s41467-021-23842-1

**Published:** 2021-05-27

**Authors:** H. Atac, M. Constantinou, Z.-E. Meziani, M. Paolone, N. Sparveris

**Affiliations:** 1 Temple University, Philadelphia, PA USA; 2 Argonne National Laboratory, Lemont, IL USA; 3 New Mexico State University, Las Cruces, NM USA

**Keywords:** Experimental nuclear physics, Theoretical nuclear physics

Correction to: *Nature Communications* 10.1038/s41467-021-22028-z, published online 19 March 2021.

The original version of the Supplementary Information associated with this Article omitted a reference to previous work in *Phys. Rev*. **C83**, 055203 (2011). This has been added as reference [34] in Section 3 of the Supplementary Information: “The Galster is a long standing phenomenological parametrization that could adequately describe the early G^n^_E_ data, but as recent experiments revealed^34^ it does not have sufficient freedom to accommodate reasonable values of the radius, without constraining or compromising the fit”.

This has been corrected in the PDF and HTML versions of the Article.

## Supplementary information

Updated Supplementary information

