# Supplementary material for: Measurement of the neutron charge radius and the role of its constituents
Source: Nat Commun. 2021 Mar 19;12:1759. doi: 10.1038/s41467-021-22028-z (PMC7979702; doi:10.1038/s41467-021-22028-z)
Supplement: Supplementary file 1 — Supplementary Information [file 41467_2021_22028_MOESM1_ESM.pdf]

Supplementary Information

Measurement of the neutron charge radius and the role of its constituents

H. Atac[1], M. Constantinou[1], Z.-E. Meziani[2,1], M. Paolone[3], and N. Sparveris[1,*]

[1]Temple University, Philadelphia, PA 19122, USA
[2]Argonne National Laboratory, Lemont, IL 60439, USA
[3]New Mexico State University, Las Cruces, NM 88003, USA
[*]e-mail: sparveri@temple.edu

Table of Contents

Section		Page
1	Description of the quadrupole transition form factor measurements	3
2	Extraction of G_E^n	3
2.1	Analysis within SU(6)	4
2.2	Analysis within large-N_c	7
3	Neutron charge radius extraction	9
3.1	Extraction from fits within a limited low-Q^2 range	10
3.2	Extraction including additional data at higher Q^2	12
3.2.1	Considerations on the CLAS data uncertainties	14
4	Flavor dependent charge densities	15
5	Lattice QCD results	15

In this document, we present details regarding to the analysis steps and the results reported in this work. More specifically we discuss: the experimental data of the quadrupole transition form factors and the nature of their uncertainties that enter into the G_E^n extraction; the steps for the G_E^n extraction from the transition form factors; the extraction procedure for $\langle r_n^2 \rangle$; the extraction of the flavor dependent form factors, densities, and mean square radii; the Lattice QCD results. Detailed tables with all the parametrizations and the final results are given, as well as additional figures that are constructive towards the discussion of this work.

1 Description of the quadrupole transition form factor measurements

The neutron electric form factor G_E^n has been extracted from measurements of the $N \to \Delta$ quadrupole transition form factors in the four-momentum transfer squared range $Q^2 = 0.04$ (GeV/c)^2 to $Q^2 = 0.20$ (GeV/c)^2. The main aspects of these measurements that are relevant within the context of the current work are briefly described in this section, and for an extensive description of the experimental details we refer to[1–5]. The measurements involve a coordinated experimental program, at JLab (Hall A) and at MAMI (A1), where:

- The experiments shared the same methodology, experimental setup specifications, and analysis tools and procedures.
- The two experimental setups offer complementarity in their kinematical coverage of momentum transfer, and the measurements were coordinated accordingly so that the low-Q^2 region is optimally measured.
- Measurements at common kinematics have been performed (i.e. at $Q^2 = 0.13$ (GeV/c)^2 see Refs.[1,3]) so that a cross check between the results from the two experimental setups is made, and consistency is ensured.
- A first measurement through the $p(e,e'p)\gamma$ channel[5] has been performed so that a comparison to the pion-electroproduction results[1–4] can be made. Such a comparison offers critical input towards the model uncertainty of the world data. For optimal comparison, the measurements were performed at the same Q^2 and with the same experimental setup for both channels.

More specifically, in both experiments an electron beam with energy $E \approx 1$ GeV has been employed on a liquid hydrogen target, and two high resolution spectrometers are used to detect protons and electrons in coincidence for the measurement of the pion electro-production excitation channel. Both setups offer a spectrometer momentum resolution of 10^{-4}, and a coincidence-time resolution between the proton and the electron spectrometers of ≈ 1 *ns*. The experimental setup offers high resolution i.e. the spectrometers focus within a very well defined bin of the kinematical phase space, and high precision cross section measurements are performed sequentially until the extended phase space has been covered (as opposed to different techniques where e.g. detectors with larger (4π) acceptance coverage but of reduced resolution are employed[6]).

An important aspect of this experimental program is the consistent, and extensive, treatment of the non-resonant pion electro-production amplitudes that interfere with the extraction of the resonant amplitudes in the $N \to \Delta$ transition. These interfering contributions, small in magnitude but large in number, can not be sufficiently constrained by the experimental measurements, and they thus result into a model uncertainty for the quadrupole transition form factors. In the past these contributions have been frequently poorly studied and rarely quoted as an uncertainty. Here, the effect of these amplitudes has been studied in the following manner:
(a) State of the art theoretical pion electroproduction models[7–11] have been employed in the data analysis. The models offer different descriptions for the background amplitudes, leading to deviations in the extracted values of the transition form factors that are quantified as a model uncertainty. This uncertainty is in turn appropriately treated in the extraction of the G_E^n.
(b) The above model uncertainties are determined within the pion electroproduction framework. However, the excitation can also be studied through the weak $p(e,e'p)\gamma$ channel. In this case the same physics signal can be extracted within a different theoretical framework, thus offering an ideal cross-check to the model uncertainties associated with the pion electroproduction channel. The branching ratio of the photon channel is very small (0.6%), two orders of magnitude smaller compared to the pion-electroproduction, and as such it was not studied until recently. To that end, the first such measurement was conducted at MAMI (A1)[5]. Measurements at the same Q^2, utilizing the same experimental setup, were performed. The results were found in agreement between the two channels[4,5], thus giving credence to the quantification of the model uncertainties.

2 Extraction of G_E^n

The G_E^n is extracted from the quadrupole transition form factor measurements[1–5] utilizing the form factor relations[12,13] determined within the SU(6) and the large-N_c frameworks. The data are analyzed independently within the two frameworks. The weighted average of the two values leads to the final G_E^n and the variance of the two values is assigned as a G_E^n theoretical

uncertainty, that is accounted for accordingly in the r_n extraction. In order to extract G_E^n from the G_E^n/G_M^n we utilize a parametrization of the well known G_M^n (as typically done in such cases, e.g.[14,15] etc). In this work we have used the recent parametrization[16]. The uncertainties associated with the G_E^n extraction are the following:

- Experimental (statistical and systematic) uncertainties of the quadrupole amplitudes.
- Model uncertainties of the quadrupole amplitudes (not applicable to measurement[5]).
- The theoretical uncertainty associated with the relations in[12,13].
- The uncertainty due to the G_M^n parametrization.

The uncertainty introduced by G_M^n has been studied by employing different G_M^n-parametrizations to the G_E^n extraction. For that we have used the most recent[16], the widely adopted in the past[17], as well as a parametrization that we worked out towards that end. The overall effect to G_E^n has been quantified $\approx 0.5\%$, and is rather small compared to the total uncertainty.

2.1 Analysis within SU(6)

A consequence of the SU(6) spin and flavor symmetry group in which the nucleon and the Δ resonance belong leads to the following expression[12]

$$\frac{G_E^n(Q^2)}{G_M^n(Q^2)} = \frac{Q}{|\mathbf{q}|}\frac{2Q}{M_N}\frac{1}{n_b(Q^2)}\frac{C2}{M1}(Q^2) \tag{1}$$

where $|\mathbf{q}|$ is the virtual photon three-momentum transfer magnitude in the γN center of mass frame, M_N is the nucleon mass, and n_b describes three-quark current terms that slightly increase the C2/M1 ratio (or correspondingly decrease the G_E^n/G_M^n), an SU(6) symmetry breaking correction that has been theoretically quantified to $\approx 10\%$[12] (i.e. $n_b \approx 1.1$).

The data have been analyzed in two ways:

i) Following the most conservative path, a theoretical uncertainty can be assigned that is equal to the full magnitude of the symmetry breaking contributions i.e. $n_b = 1.1 \pm 0.1$. Considering the confidence with which the underlying theory is able to determine the level of the symmetry breaking contributions, the above assumption leads to a safe estimation, and most likely to an overestimation, of the theoretical uncertainty.

ii) The wealth of the experimental world data for $C2/M1$[1–6,18–20] and for G_E^n/G_M^n[14,15,21–32] allow to determine the magnitude of the symmetry breaking corrections [1]. Refinements to the corrections are implemented utilizing the LQCD data for G_E^n/G_M^n reported in this work (see later in the LQCD section).

The results from (i) and (ii) are in excellent agreement; the difference between the analysis (i) and (ii) results to a very small effect in the r_n extraction (see the radius extraction section). This comes as a consequence of the confirmation of the n_b theoretical prediction[12] by the experimental data (see Fig. 1). The experimental determination of the breaking corrections n_b takes place as follows:

First, we determine the set of appropriate functions $F_R(Q^2)$ that can successfully parametrize the G_E^n/G_M^n ratio. To that end we have identified the following forms that are able to provide a good fit to the data:

$$F_R(x) = \sum_{i=1}^{j} a_i x_i^i, \quad F_R(x) = \frac{\sum_{i=1}^{j} b_i x_i^i}{(1+\sum_{j=1}^{j} c_j x_j^j)}, \quad F_R(x) = (1-\exp^{(d_1 x)}).$$

For each one of these functions:

- The $F_R(Q^2)$ parametrization is determined by fitting to the G_E^n/G_M^n world data[14,15,21–32].
- The $F_R^*(Q^2)$ is determined by fitting to the G_E^n/G_M^n ratios as derived from the $N \to \Delta$ measurements[1–6,18–20] through Eq. 1 for n_b=1.
- The breaking corrections are then determined, for each functional form, through: $n_b(Q^2) = F_R^*(Q^2)/F_R(Q^2)$.

[1] A revised analysis of the[2] has updated the C2/M1 result to $(-4.10 \pm 0.27_{stat+sys} \pm 0.26_{mod})\%$

	$\sum_{i=1}^{i} a_i x_i^i$		$\frac{\sum_{i=1}^{i} b_i x_i^i}{(1+\sum_{j=1}^{j} c_j x_j^j)}$				$(1-\exp^{(d_1 x)})$
$F_{\mathrm{R}}(x)$							
a_1	1.420	1.670	-	-	-	-	-
a_2	-2.351	-5.962	-	-	-	-	-
a_3	-	10.968	-	-	-	-	-
b_1	-	-	1.679	16.050	1.790	11.236	-
b_2	-	-	14.840	11.590	20.340	0.802	-
b_3	-	-	-	15.168	0.801	15.168	-
b_4	-	-	-	-	-	43.971	-
c_1	-	-	13.602	43.980	18.111	1.756	-
c_2	-	-	31.396	1.757	49.377	16.089	-
c_3	-	-	-	-	-40.446	-	-
d_1	-	-	-	-	-	-	-0.336
$F^{*}_{\mathrm{R}}(x)$							
a_1	1.587	1.707	-	-	-	-	-
a_2	-2.243	-4.349	-	-	-	-	-
a_3	-	6.078	-	-	-	-	-
b_1	-	-	1.851	0.170	1.919	12.346	-
b_2	-	-	2.495	15.730	23.081	0.800	-
b_3	-	-	-	3.455	0.801	15.369	-
b_4	-	-	-	-	-	35.134	-
c_1	-	-	6.090	15.298	18.943	1.904	-
c_2	-	-	-0.955	1.793	34.286	17.579	-
c_3	-	-	-	-	-12.404	-	-
d_1	-	-	-	-	-	-	-0.379
$F^{1}_{\mathrm{R}}(x)$							
a_1	1.240	1.332	-	-	-	-	-
a_2	-2.102	-3.833	-	-	-	-	-
a_3	-	6.228	-	-	-	-	-
b_1	-	-	1.363	1.774	0.996	9.800	-
b_2	-	-	2.816	11.497	27.413	0.801	-
b_3	-	-	-	3.934	0.802	15.753	-
b_4	-	-	-	-	-	48.822	-
c_1	-	-	5.882	20.740	16.773	1.366	-
c_2	-	-	4.267	1.326	58.324	16.228	-
c_3	-	-	-	-	254.194	-	-
d_1	-	-	-	-	-	-	-0.296

Table 1. The fitted parameters of the $F_{\mathrm{R}}(x)$, $F^{*}_{\mathrm{R}}(x)$, and $F^{1}_{\mathrm{R}}(x)$ functions.

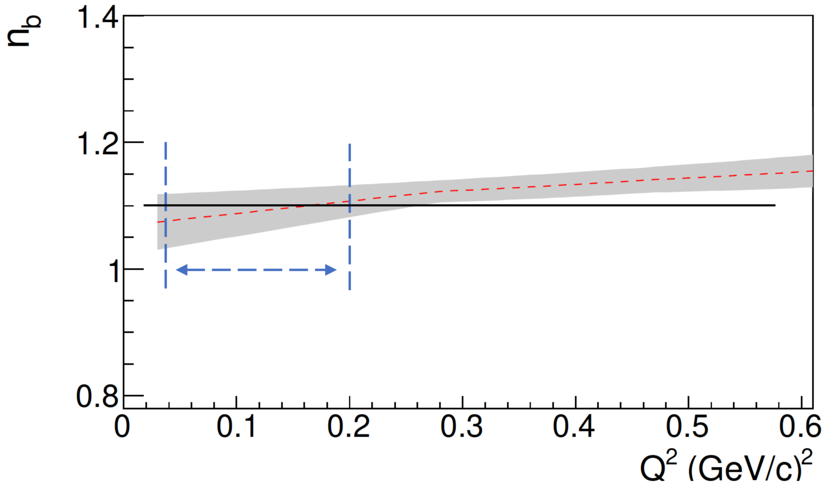

Figure 1. The breaking corrections n_b (dashed line) and δn_b uncertainty (shaded band) at the 1σ or 68% confidence level. The solid line indicates the n_b as theoretically determined in[12]. The horizontal double-arrow marks the Q^2-range where the corrections have been employed for the measurement of G_E^n in this work.

- The procedure is then repeated for all appropriate functions.
- We combine all of the above $n_b(Q^2)$ results (i.e. as derived for all the functional forms) and we experimentally determine the $n_b(Q^2) \pm \delta n_b(Q^2)$: The $n_b(Q^2) = [n_{b(max)}(Q^2) + n_{b(min)}(Q^2)]/2$, where $n_{b(max)}$ and $n_{b(min)}$ are the maximum and minimum n_b derived at a given Q^2. The $\delta n_b(Q^2)$ results from the maximum spread of the $n_b(Q^2)$ solutions at any given Q^2, i.e. $[n_{b(max)}(Q^2) - n_{b(min)}(Q^2)]$.

Since the neutron data do not extend lower than $Q^2 = 0.14$ (GeV/c)^2 we have decided to explore refinements in the determination of n_b by utilizing state of the art LQCD calculations for the G_E^n/G_M^n ratio that extend lower in Q^2. The LQCD results bring valuable input in describing the Q^2-dependence of G_E^n/G_M^n in this region based on ab-initio QCD calculations, thus having the potential to drive more accurately the fits. The procedure followed here is the following:

- First, the LQCD G_E^n/G_M^n results are normalized to the neutron world data, within the region where there is overlap for both data sets i.e. $Q^2 \geq 0.14$ (GeV/c)^2. The LQCD results agree remarkably well to the experimental data but we nevertheless introduce this normalization in order to absolutely baseline the two data sets within the Q^2 range that they overlap.
- The $F_R^l(k)$ is then determined by fitting the LQCD data to the same set of functions, as in the previous step, for the full momentum transfer range of the LQCD data-set so that the data at $Q^2 < 0.14$ (GeV/c)^2 are included.
- The breaking corrections are now given by: $n_b(k) = F_R^*(k)/F_R^l(k)$

For $Q^2 \geq 0.14$ (GeV/c)^2 the corrections are naturally identical to the ones determined using the experimental neutron data, since there the LQCD ratios have been normalized to the experimental data. For the lower Q^2 region, the resulting refinement to the n_b determination is $\leq \pm 0.3\%$ i.e. $\delta n_b(Q^2) \leq \pm 0.003$. That is an order of magnitude smaller than the total experimental δn_b uncertainty, that ranges between $\pm 2.5\%$ to $\pm 4\%$ in that region. Furthermore, we do not allow these refinements to reduce the δn_b uncertainty in any way, but only to increase it where the results extend it further than what was determined using the experimental data only. The fitted parameters for the $F_R(x)$, $F_R^*(x)$, and $F_R^l(x)$ are given in Table 1. The experimentally determined $n_b(Q^2)$ is shown in Fig. 1 and the shaded band corresponds to the n_b uncertainty. The solid line marks the n_b as theoretically determined in[12], and one can note the remarkable agreement between the theoretical and the experimental values. The n_b results are also listed in Table 2 for the Q^2 range that they have been used for the G_E^n extraction. The G_E^n results and the breakdown of the uncertainties are given in Table 3.

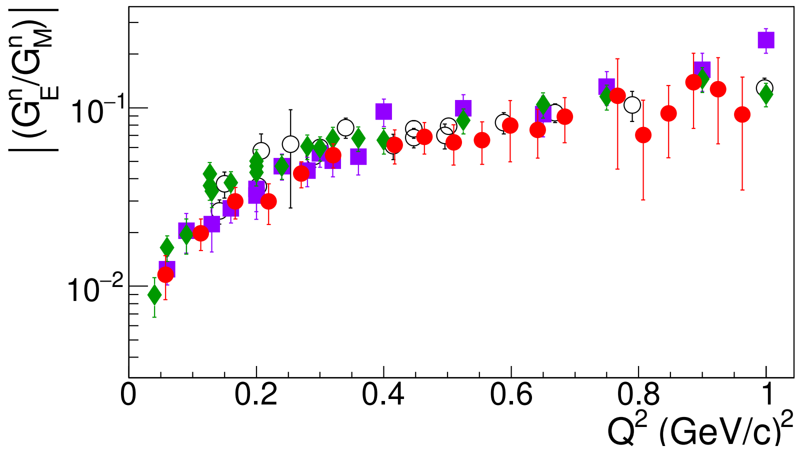

Figure 2. The elastic neutron form factor ratio from the large-N_c analysis.
The G_E^n/G_M^n results from the large-N_c analysis with the Coulomb quadrupole data (filled diamonds) and with the Electric quadrupole data (filled boxes) from measurements[1–6, 18–20]. The neutron world data (open-circles) and the LQCD results (filled circles) are also shown. The error bars correspond to the total uncertainty, at the 1σ or 68% confidence level.

Q^2 (GeV/c)^2	n_b	$n_{b(min)}$	$n_{b(max)}$
0.040	1.076	1.034	1.119
0.050	1.078	1.037	1.120
0.060	1.080	1.040	1.120
0.070	1.082	1.043	1.121
0.080	1.084	1.046	1.122
0.090	1.086	1.049	1.123
0.100	1.088	1.052	1.124
0.110	1.090	1.055	1.125
0.120	1.092	1.058	1.125
0.130	1.093	1.061	1.126
0.140	1.095	1.064	1.127
0.150	1.097	1.067	1.128
0.160	1.099	1.070	1.129
0.170	1.101	1.073	1.130
0.180	1.103	1.076	1.131
0.190	1.105	1.079	1.132
0.200	1.107	1.082	1.132

Table 2. The n_b corrections as experimentally determined in the region of the latest G_E^n measurements.

2.2 Analysis within large-N_c

The relation between the G_E^n and the quadrupole transition form factors has also been established through large-N_c relations[13]. The relations take the form

$$\frac{E2}{M1}(Q^2) = \left(\frac{M_N}{M_\Delta}\right)^{3/2} \frac{M_\Delta^2 - M_N^2}{2Q^2} \frac{G_E^n(Q^2)}{F_2^p(Q^2) - F_2^n(Q^2)} \tag{2}$$

$$\frac{C2}{M1}(Q^2) = \left(\frac{M_N}{M_\Delta}\right)^{3/2} \frac{Q_+ Q_-}{2Q^2} \frac{G_E^n(Q^2)}{F_2^p(Q^2) - F_2^n(Q^2)} \tag{3}$$

where $F_2^{p(n)}$ are nucleon's Pauli form factors, M_Δ is the mass of the Δ, and $Q_\pm = ((M_\Delta \pm M_N)^2 + Q^2)^{\frac{1}{2}}$. Here the experimental data base is extended to include the Electric quadrupole (E2) transition, which in turn allows for an improved extraction of the

$Q^2 (\mathrm{GeV}/c)^2$	G_E^n	$\delta G^n_{E(exp)}$	$\delta G^n_{E(mod)}$	$\delta G^n_{E(n_b)}$
0.040	0.0128	0.0011	0.0034	0.0005
0.060	0.0222	0.0015	0.0014	0.0008
0.090	0.0242	0.0014	0.0055	0.0008
0.127	0.0420	0.0049	0.0024	0.0013
0.130	0.0388	0.0015	0.0065	0.0012
0.200	0.0460	0.0025	0.0027	0.0010
0.200	0.0397	0.0090	-	0.0009

Table 3. The neutron electric form factor derived within the SU(6) analysis. The G_E^n results with their experimental and model uncertainties, as described in the text (with the uncertainty δn_b constrained by the experimental form factor world data). The last data point, at $Q^2 = 0.20\ (\mathrm{GeV}/c)^2$, involves the measurement from $p(e,e'p)\gamma$[5] and it thus does not have a model uncertainty associated with the pion electroproduction background amplitudes.

$Q^2 (\mathrm{GeV}/c)^2$	G_E^n	$\delta G^n_{E(exp)}$	$\delta G^n_{E(th)_1}$
0.040	0.0151	0.0010	0.0023
0.060	0.0246	0.0011	0.0037
0.090	0.0285	0.0012	0.0043
0.127	0.0489	0.0040	0.0073
0.130	0.0447	0.0013	0.0067
0.200	0.0525	0.0021	0.0079
0.200	0.0459	0.0074	0.0069

Table 4. The neutron electric form factor derived from the large-N_c analysis. The neutron electric form factor derived from the large-N_c analysis of the Electric and the Coulomb quadrupole amplitude measurements.

G_E^n. For the well known G_E^p, G_M^p and G_M^n, that enter in the expressions through the Pauli form factors, we have used recent parametrizations (see next sections for details).

The above relations come with a 15% theoretical uncertainty[13] that has been accounted for in the G_E^n extraction. That level of uncertainty is further supported by a third relation, also derived in the same work, that connects the isovector Pauli FF to the $N \to \Delta$ magnetic dipole FF[13]; that relation has been shown to work well at the 13% level. The level of theoretical uncertainty can be further cross checked using experimental data, when one extracts the G_E^n independently through the Coulomb and the Electric quadrupole transitions. The results from the two different analyses validate the 15% level of agreement, as can be seen in Fig. 2. We have thus adopted this number as a theoretical uncertainty in the data analysis.

From each experiment we derive one G_E^n value, from both the measurements of the Coulomb and of the Electric quadrupole form factor by taking the weighted average of the two independently derived G_E^n values. The results are given in Table 4. The results are in great agreement with that of the SU(6) analysis. The weighted average of the large-N_c and of the SU(6) results leads to the final G_E^n value. The variance of the two values is assigned as a G_E^n theoretical uncertainty, and is accounted for accordingly in the r_n extraction. The final results for G_E^n are given in Table 5.

$Q^2 (\mathrm{GeV}/c)^2$	G_E^n	δG_E^n	$\delta G^n_{E(th)_2}$
0.040	0.0143	0.0020	0.0012
0.060	0.0228	0.0019	0.0012
0.090	0.0269	0.0035	0.0021
0.127	0.0442	0.0047	0.0034
0.130	0.0417	0.0048	0.0030
0.200	0.0471	0.0035	0.0033
0.200	0.0425	0.0067	0.0031

Table 5. Final results for the neutron electric form factor. The G_E^n final results as derived from the SU(6) and the large-N_c analysis. The theoretical uncertainty is derived from the variance of the results from the two analyses.

3 Neutron charge radius extraction

The neutron mean square charge radius is related to the slope of the neutron electric form factor as $Q^2 \to 0$ through

$$\langle r_{\rm n}^2 \rangle = -6 \frac{dG_{\rm E}^{\rm n}(Q^2)}{dQ^2}\Big|_{Q^2\to 0}. \quad (4)$$

The $G_{\rm E}^{\rm n}(Q^2)$ has to be parametrized and fitted to the experimental data, and from the slope at $Q^2 = 0$ the $\langle r_{\rm n}^2 \rangle$ is determined. The data derived in this work offer the level of precision, and the Q^2-range, that allow to explore for suitable $G_{\rm E}^{\rm n}(Q^2)$ functional forms with an additional free parameter compared to what was possible in the past (i.e. with three free parameters). In turn, this allows to extract the $\langle r_{\rm n}^2 \rangle$ from the $G_{\rm E}^{\rm n}$ measurements, which has not done before. We have explored various functional forms in order to identify those appropriate, in the following way:

i) The functions have been fitted to the $G_{\rm E}^{\rm n}$ results reported in this work (data-set in Table 5) and to the world data[14, 15, 21–32].

ii) For the functions that offer a good fit to the data, further tests are performed with pseudo-data; these tests aim to place the parametrizations under stress in terms of their limitations to fits that engage data of higher precision and of extended kinematical coverage.

iii) The $\langle r_{\rm n}^2 \rangle$ extraction is repeated with multiple $G_{\rm M}^{\rm n}$ parametrizations so that the uncertainty introduced by the $G_{\rm M}^{\rm n}$ parametrization is quantified; for that part we have found ± 0.0009 (fm^2), an order of magnitude smaller compared to the total $\langle r_{\rm n}^2 \rangle$ uncertainty.

Our studies have shown that the most robust function for the radius extraction takes the form

$$G_{\rm E}^{\rm n}(Q^2) = (1 + Q^2/A)^{-2} \frac{B\tau}{1 + C\tau}, \quad (5)$$

where $\tau = Q^2/4m^2$, and A, B, C are free parameters. It involves a similar form to the Galster[33]. The Galster is a long standing phenomenological parametrization that could adequately describe the early $G_{\rm E}^{\rm n}$ data, but as recent experiments revealed[34] it does not have sufficient freedom to accommodate reasonable values of the radius, without constraining or compromising the fit. Here, instead of using the standard dipole form factor with $\Lambda^2 = 0.71(\mathrm{GeV}/c)^2$ an additional free parameter A is introduced (see Eq. 5). Our fit to the data gives $\langle r_{\rm n}^2 \rangle = -0.110 \pm 0.008$ (fm^2) with a reduced χ^2 of 0.74. The fitted parameters are given in Table 6.

A second parametrization, giving a good fit to the data, involves the sum of two dipoles

$$G_{\rm E}^{n}(Q^2) = \frac{A}{(1+\frac{Q^2}{B})^2} - \frac{A}{(1+\frac{Q^2}{C})^2}. \quad (6)$$

This form has been explored in the past[15] but with only two free parameters and with the $\langle r_{\rm n}^2 \rangle$ already constrained by the measurement of the neutron-electron scattering length. This fit (see Table 6) exhibits an excellent agreement to the one of Eq. 5. The two curves are nearly indistinguishable by eye as seen in Fig. 3a. Considering the weighted average of the two methods we derive a nearly identical result of $\langle r_{\rm n}^2 \rangle = -0.110 \pm 0.007$ (fm^2). Nevertheless, our pseudo-data studies showed that the two-dipole fit suffers from limitations in the determination of the $G_{\rm E}^{\rm n}$-slope. We thus adopt only the paramterization of Eq. 5 and quote it's fitted result $\langle r_{\rm n}^2 \rangle = -0.110 \pm 0.008$ (fm^2) as our final result for the neutron charge radius.

Parametrization	A	B	C
Param. 5	0.505 ± 0.079	1.655 ± 0.126	0.909 ± 0.583
Param. 6	0.130 ± 0.039	1.790± 0.409	0.419 ± 0.0980

Table 6. Fitted parameters for the two $G_{\rm E}^{\rm n}$ parametrizations.

We have also explored the radius extraction by adopting the scenario where the uncertainty of the symmetry breaking corrections in the SU(6) analysis is treated very conservatively i.e. $n_{\rm b} = 1.1 \pm 0.1$. In this case the final result becomes $\langle r_{\rm n}^2 \rangle = -0.109 \pm 0.009$ (fm^2) with a reduced χ^2 of 0.74. This scenario tends to overestimate the uncertainty of the symmetry breaking terms, but as this is not a dominant factor in the radius extraction the $r_{\rm n}$-uncertainty is not affected significantly.

The particle data group (PDG) average value, $\langle r_{\rm n}^2 \rangle = -0.1161 \pm 0.0022$ (fm^2), is the weighted average of five values[35–38] based on the measurement of the neutron-electron scattering length. One of these measurements[37] disagrees with [35,36], but as this disagreement has not been resolved the value from measurement[37] is considered in the PDG average, for the time being. Our result, $\langle r_{\rm n}^2 \rangle = -0.110 \pm 0.008$ (fm^2), comes to update the world average value for the neutron charge radius. The recent measurement, like the ones from[35,36], also disagrees with that of [37], and considering that this disagreement comes from a

different method for the radius extraction one may want to exclude the value of[37] from the world data average. The latest weighted average value, including our measurement and excluding the one of[37], becomes $\langle r_{\rm n}^2 \rangle = -0.1152 \pm 0.0017$ (fm^2) and is shown in Fig. 3b. In the current PDG world-data average, the uncertainty that is calculated following the standard weighted least-squares procedure is further enhanced by a factor of $S = [\chi^2/(N-1)]^{1/2}$ due to the discrepancies that are introduced from the inclusion of the Alexandrov'86 measurement in the data-set. In the most recent world-data average, with the inclusion of the new measurement and the subsequent exclusion of the Alexandrov'86, these discrepancies are raised and the term S does not contribute any further to the world-data average uncertainty. Effectively, the biggest benefit to the uncertainty results from the resolution of the discrepancies in the $\langle r_{\rm n}^2 \rangle$ measurements that the current work allows for. For the completeness of this discussion, we can also comment that if one considers in the world-data average both the new $\langle r_{\rm n}^2 \rangle$ measurement as well as that of Alexandrov'86, then the uncertainty improves by 10%, from ± 0.0022 (fm^2) to ± 0.0020 (fm^2). Nevertheless, the results of this work suggest that the Alexandrov'86 has to be eliminated from the world-data average in which case the uncertainty is further reduced to ± 0.0017 (fm^2).

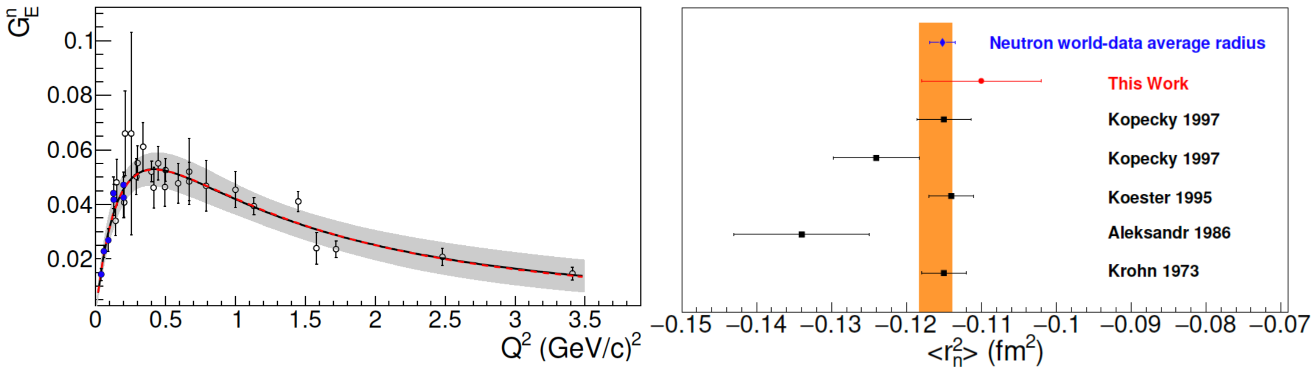

Figure 3. Left panel: The G_E^n results from this work (filled-circles) and the G_E^n world data (open-circles)[14, 15, 21–32]. The error bars correspond to the total uncertainty, at the 1σ or 68% confidence level. The solid (black) curve shows the fit to the data from the parametrization of Eq. 5 with its uncertainty (shaded band). The dashed (red) line shows the fit from parametrization of Eq. 6. The two fits practically overlap. Right panel: The $\langle r_{\rm n}^2 \rangle$ measurement from this work and from the references[35–38] that are currently included in the PDG $\langle r_{\rm n}^2 \rangle$ analysis. The orange-band marks the PDG averaged $\langle r_{\rm n}^2 \rangle$ value. The recent weighted average of the world data is also shown, when the $\langle r_{\rm n}^2 \rangle$ measurement reported here is included in the calculation.

3.1 Extraction from fits within a limited low-Q^2 range

The extraction of the neutron charge radius has been performed by fitting over an extended Q^2 range, following the experience from the extraction of the proton charge radius, where the world data base is far more extensive compared to the one of the neutron. In that case, the charge radius extraction has been traditionally based on fits of functional forms over an extended kinematic Q^2 range (e.g. such as in the most recent r_p extraction from the Mainz G_E^p measurements, etc). Fitting over a limited low Q^2-range in order to extract the proton radius was first attempted very recently, at the PRad experiment. In that case though the G_E^p was accessed at much lower momentum transfers compared to the neutron measurements, namely in the range $Q^2 = 0.0002$ (GeV$/c$)^2 to $Q^2 = 0.06$ (GeV$/c$)^2.

One consideration here is that the proton and the neutron electric form factor parametrizations are inherently different (i.e. the G_E^n changes slope and follows a monotonic fall-off at low Q^2). With that in mind we here explore the potential of G_E^n fits that are limited only in the low Q^2 region. The global fit for G_E^n shows that a maximum is reached shortly after $Q^2 = 0.4$ (GeV$/c$)^2. We thus limit our studies within the range [0 - 0.4 (GeV$/c$)^2] that G_E^n is expected to be monotonic. In doing so one has to consider to following factors: what is the choice of the functional forms and the number of parameters that will be fitted, what is the optimal fitting range, and how does the extracted radius depend on the ansatz for the fit model. Here we follow a procedure that has been frequently adopted for the extraction of the proton charge radius. We have studied a variety of functions so that we can identify those that are appropriate for the fit to the data. The functional forms that allow the extraction of the charge radius with a meaningful precision are divided in two groups. The first group is based on polynomials and involves polynomials with varying orders and combinations of polynomials with a dipole. The stability of the extracted radius is observed within the group by using polynomials of different degree, while the variance of the fitted values indicates the model uncertainty of the

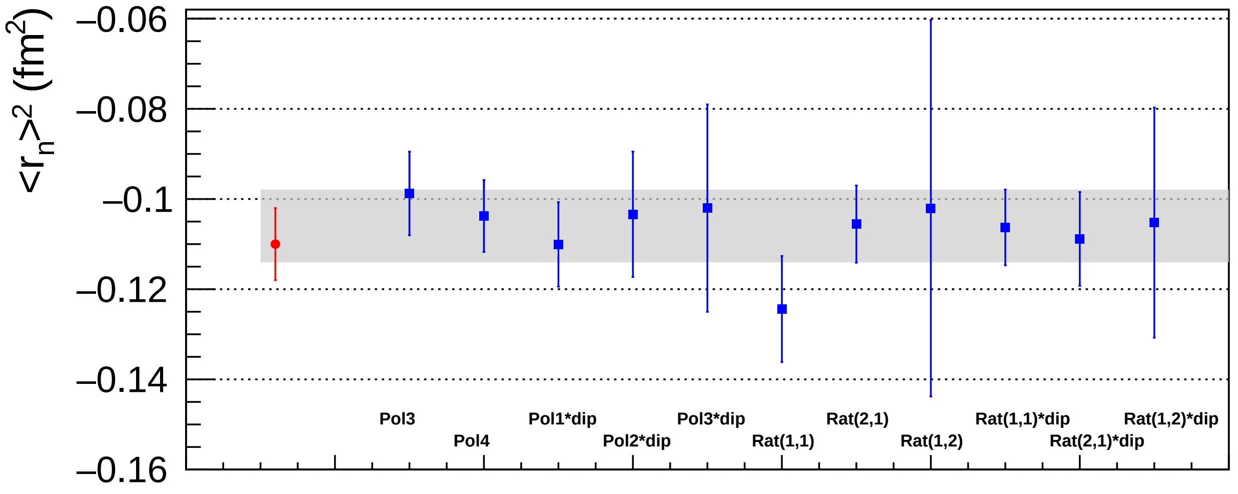

Figure 4. The $\langle r_{\rm n}^2\rangle$ as extracted with the different model functions (blue boxes) from fits within the low Q^2 range. The error bars correspond to the total uncertainty, at the 1σ or 68% confidence level. The gray band marks the final result, from the combination of all the fits of both groups of functions (see text for details as well as Tab. 7). The red point marks the final result from the fit over the extended Q^2 range, namely $\langle r_{\rm n}^2\rangle = -0.110 \pm 0.008$ (fm^2).

group. The second group is based on rational functions of the form

$$Rat(i,j)(Q^2) = \frac{\sum_{i=1}^{n} \alpha_i Q^{2i}}{1+\sum_{j=1}^{m} \beta_j Q^{2j}}$$

and combinations of Rat(n,j) with dipole. These groups of functions have been utilized in the past for the proton radius extraction. Other functional forms that we explored (e.g. exponential-based functions $f(Q^2) = \sum_{i=0}^{n} \alpha_i Q^{2i}(1-e^{-\beta Q^2})$, etc) give consistent results for the charge radius but with large uncertainties and as such we do not consider them here. When it comes to the number of fitted parameters, n_p, one has to make sure that n_p is not too small since in such a case the data will not be properly reproduced, while at the same time one has to avoid a very high level of flexibility in order to avoid erratic fits. For example, in the polynomial fits we have found that starting with a 3rd order polynomial we can have a good fit, but after the 5th order polynomial the fitted function follows an erratic, non-monotonic behavior through the data points at the higher end of the Q^2 fitting range. In the latter case, the fitted value for the $\langle r_{\rm n}^2\rangle$ is found to be consistent to that of the fits with the lower order polynomials. Nevertheless, for consistency we have decided to exclude such fits from the determination of the charge radius since our requirement is to fit a monotonic function at low momentum transfers. Polynomials$\times$dipole were able to provide a good fit, contrary to polynomial+dipole that would result into non-stable, erratic fits. Functional forms within the group were not considered when their resulting uncertainties were too large to contribute meaningfully to the determination of the charge radius, such as rational functions higher than Rat(2,2). The $\langle r_{\rm n}^2\rangle$ results from the individual fits within the two groups are shown in Fig. 4. The comparison of all the G_E^n fits for the functions within the two groups are shown in Fig. 5.

	[0 - 0.3 (GeV/c)^2]	[0 - 0.4 (GeV/c)^2]
Polynomial group	$\langle r_{\rm n}^2\rangle = -0.105 \pm 0.006 \pm 0.002_{\rm mod}$ (fm^2)	$\langle r_{\rm n}^2\rangle = -0.104 \pm 0.005 \pm 0.003_{\rm mod}$ (fm^2)
Rational group	$\langle r_{\rm n}^2\rangle = -0.108 \pm 0.006 \pm 0.002_{\rm mod}$ (fm^2)	$\langle r_{\rm n}^2\rangle = -0.108 \pm 0.006 \pm 0.002_{\rm mod}$ (fm^2)
$\langle r_{\rm n}^2\rangle = -0.107 \pm 0.006 \pm 0.002_{\rm mod} \pm 0.002_{\rm group}$ (fm^2)		

Table 7. The extracted $\langle r_{\rm n}^2\rangle$ from the fits within a low-Q^2 range.

For the charge radius, the weighted average is extracted separately for each one of the two groups. A systematic uncertainty

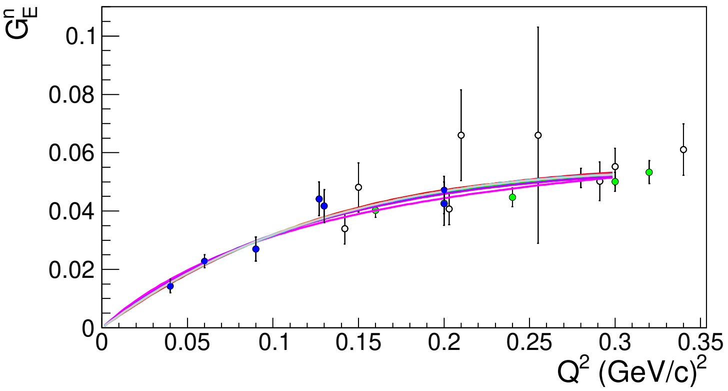

Figure 5. The low-Q^2 G_E^n fits with all the functions within the polynomial and the rational groups. The data from the analysis of the MAMI and Hall-A measurements are marked as blue points and the ones from the CLAS as green points. The error bars correspond to the total uncertainty, at the 1σ or 68% confidence level.

is also quantified within each group (i.e. a model uncertainty of the group) from the weighted variance of the results from all the fits within the group. The results from the two groups tend to have a similar total uncertainty (i.e. when the weighted average and the group's model uncertainty are combined). Nevertheless there is a small systematic difference of the two group's central values, as studies over a varying fitting range have shown. For that reason a third uncertainty is determined: here we consider the spread of the two central values as indicative of the uncertainty that is associated with the choice of the group. Therefore the final result is given by the average of these two values, while the half of the difference of the two values is assigned as an additional (group) uncertainty (see Tab. 7). This uncertainty has to be added linearly, and not quadratically, to the other two uncertainties. Lastly, the sensitivity of the results to the fitting range has been explored. There is an interplay of the fitting and of the model/group uncertainties depending on the fitting range in Q^2. Our studies have shown that as we increase the upper bound of the fitting range, Q^2_{max}, the fitting uncertainties tend to improve but at the expense of the group's model uncertainty. When all uncertainties are considered, the overall level of uncertainty for the results within the [0 - 0.3 $(\mathrm{GeV}/c)^2$] and within the [0 - 0.4 $(\mathrm{GeV}/c)^2$] range are comparable, with a very small benefit if the fits are conducted within the [0 - 0.3 $(\mathrm{GeV}/c)^2$]. As a general remark, when the overall uncertainty (i.e. from the combination of the fitting and model ones) is equivalent for two different fitting ranges it may be preferable to pursue the results through fits within the range where the model uncertainties tend to minimize, considering that the fitting uncertainties are very strictly defined and accurately determined, while the determination of model uncertainties entails a higher level of uncertainty. Lastly, limiting the fitting range even further, within the [0 - 0.2 $(\mathrm{GeV}/c)^2$] range, is not beneficial since the fitting uncertainties increase significantly. The results from the fits within each group are shown in Tab. 7. With the low-Q^2 fits we find that $\langle r_n^2 \rangle = -0.107 \pm 0.006 \pm 0.002_{\mathrm{mod}} \pm 0.002_{\mathrm{group}}$ (fm^2). This value is in excellent agreement compared to what we derive when we fit within the extended Q^2 range, namely $\langle r_n^2 \rangle = -0.110 \pm 0.008$ (fm^2). Nevertheless we conclude that the fits at low-Q^2 are not able to provide a more precise determination of $\langle r_n^2 \rangle$ compared to the fit over the extended Q^2 range, while a higher level of model uncertainty is introduced to the result by the low-Q^2 fits. We here note that the inclusion of the CLAS data in the low-Q^2 fits (see Section 3.2 that follows) reveal a further increase to the *group* uncertainty, from $\pm 0.002_{\mathrm{group}}$ (fm^2) to $\pm 0.004_{\mathrm{group}}$ (fm^2). Our studies indicate that more data are required lower in Q^2 so that these uncertainties can be further suppressed, and that the G_E^n fits over the extended Q^2 range provide the most precise and consistent measurement of $\langle r_n^2 \rangle$.

3.2 Extraction including additional data at higher Q^2

The limiting factor in the extraction of the neutron charge radius has been the lack of G_E^n data at low momentum transfers. This limitation is now raised by the latest data provided by the MAMI and the JLab/Hall-A measurements in the region $Q^2 = 0.04$ $(\mathrm{GeV}/c)^2$ to $Q^2 = 0.20$ $(\mathrm{GeV}/c)^2$. The intermediate and the high Q^2 regime on the other hand is already sufficiently covered by the G_E^n world data and the $\langle r_n^2 \rangle$ fits are not limited by the luck of data in this region.

The quadrupole transition form factor measurements extend higher in Q^2, in the region that has already been accessed by G_E^n measurements. One can thus naturally consider to extend the current analysis to higher momentum transfers so as to enrich the G_E^n data base even further, with overlapping measurements, in the hope to improve the fits for the $\langle r_n^2 \rangle$ extraction. In principle one can attempt to do that. Nevertheless, while the relations that relate the G_E^n to the quadrupole transition form factors hold on very solid ground in the low Q^2 region (i.e. the region of the MAMI and the JLab/Hall-A measurements) they tend to hold less well at high momentum transfers. There is no sharp, formally defined Q^2 cut-off-value after which these

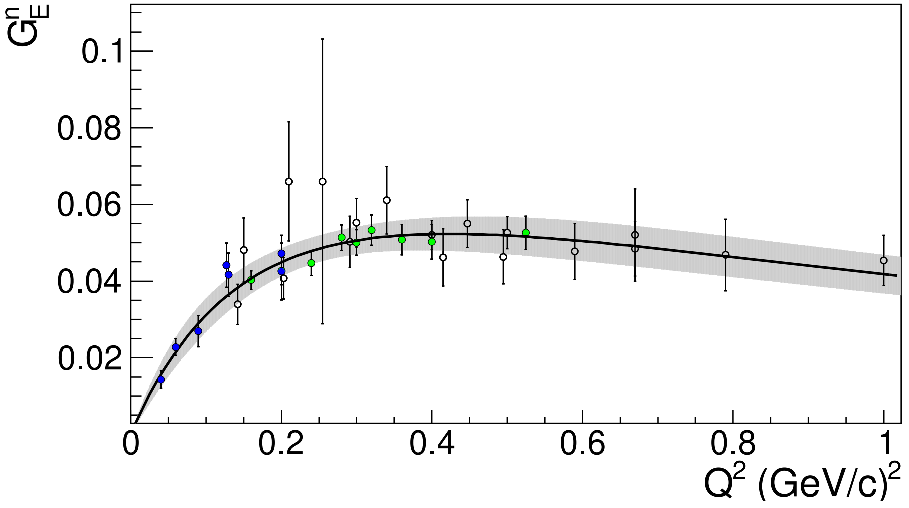

Figure 6. The G_E^n results from the MAMI and the JLab/Hall-A measurements (blue points) and from the CLAS measurements (green points). The error bars correspond to the total uncertainty, at the 1σ or 68% confidence level.

relations do not hold, and as such they should be treated with caution at increasing momentum transfers e.g. their level of theoretical uncertainty tend to increase around ≈ 1 (GeV$/c$)^2 to a level that one may not wish to risk including them in the current analysis, considering the level of precision that we are after for the charge radius extraction. One thus has to walk along a fine line, by adding measurements at intermediate momentum transfers, e.g. higher than $Q^2 = 0.20$ (GeV$/c$)^2 but not going very high in Q^2, so that a clear benefit to the r_n extraction is achieved and without compromising the extraction with theoretical uncertainties.

Here we expand the range of our analysis and we extract the G_E^n from the JLab/CLAS[6] lowest momentum-transfer data-set which involves measurements of the quadrupole transition form factors up to $Q^2 = 0.52$ (GeV$/c$)^2. The measurements are taken within a kinematic region where one can still feel comfortable applying these relations. The extracted G_E^n from these measurements is shown in Fig. 6, and the results are in excellent agreement with the G_E^n world data in the same region. When we include this additional set of data in the $\langle r_n^2 \rangle$ extraction and we fit over the complete Q^2-range of the G_E^n measurements we find a $\langle r_n^2 \rangle = -0.107 \pm 0.007$ (fm^2), compared to $\langle r_n^2 \rangle = -0.110 \pm 0.008$ (fm^2) when the CLAS data are not included. Here we observe a minor improvement to the $\langle r_n^2 \rangle$ uncertainty with the inclusion of the CLAS data, but we suggest that this result is treated with some caution. We have some concerns, that part of the reported CLAS uncertainties may be underestimated which could in turn influence / bias the level of the $\langle r_n^2 \rangle$ uncertainty. We discuss these considerations in more detail in the section that follows (Section 3.2.1).

We do not consider extending the data-set with measurements at higher Q^2 e.g. by including the CLAS data that are higher than $Q^2 \approx 0.7$ (GeV$/c$)^2, for the following reasons. Firstly, as we noted above, in such a case we would be entering a region that is associated with a high level of theoretical uncertainties. Secondly, when we investigate the effect of including the additional data up to $Q^2 = 1$ (GeV$/c$)^2 we did not observe any further benefit to the fits, namely we derive the same level of $\langle r_n^2 \rangle$ uncertainty. Thus in such a case one would risk to introduce further uncertainties of theoretical nature to the $\langle r_n^2 \rangle$ extraction without any benefit to the fitting uncertainty. Lastly, the CLAS results at $Q^2 = 1$ (GeV$/c$)^2 disagree with the results from the Hall-A experiment[20] at the same Q^2 that is based on recoil polarization measurements. The Hall-A experiment offers a nearly model-independent analysis and also utilizes a setup with superior resolution compared to the one of CLAS. If we were to consider these (disagreeing) data we could risk further bias to the $\langle r_n^2 \rangle$ extraction from the tensions among the competing transition form factors measurements in that region.

Lastly, we explore the influence of the CLAS data to the $\langle r_n^2 \rangle$ extraction from the fits within a limited low-Q^2 range, as discussed in Section 3.1. The results for the fitted functions when the CLAS data are included are shown in Fig. 7 and they are compared to the results of the fits when the CLAS data are not included in the analysis. The final results are summarized in Tab. 8. Looking at the results we are able to make a number observations and to draw a set of conclusions with regard to the strength of the low-Q^2 fits. First, we note that the results of the polynomial group maintains remarkable stability compared to the analysis when the CLAS data are not included. The central value of the rational group, on the other hand, observes a shift compared to the analysis when the CLAS data are not included. This effectively enhances the group uncertainty from

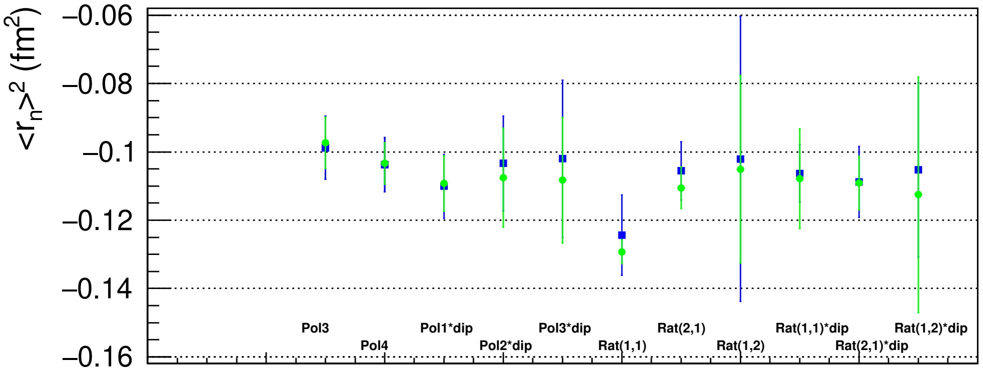

Figure 7. The $\langle r_n^2 \rangle$ as extracted with the different model functions from fits within the low Q^2 range. The results are shown with the inclusion of the CLAS data (green circles) and without (blue boxes). The error bars correspond to the total uncertainty, at the 1σ or 68% confidence level.

$\pm 0.002_{\text{group}}$ (fm^2) to $\pm 0.004_{\text{group}}$ (fm^2) when the CLAS data are included. Another observation is that the model uncertainty within each group tends to also increase when we fit in the [0 - 0.4 $(\text{GeV}/c)^2$] range, but not at the [0 - 0.3 $(\text{GeV}/c)^2$] range. One can conclude that for low-Q^2 fits within that region we are rather sensitive to model uncertainties that depend on the choice of the fitted parametrization. These uncertainties tend to be comparable to the statistical uncertainty of the fits. These uncertainties can be further suppressed when more data are acquired at lower momentum transfers and the fitting range can be limited even lower in Q^2.

	[0 - 0.3 $(\text{GeV}/c)^2$]	[0 - 0.4 $(\text{GeV}/c)^2$]
Polynomial group	$\langle r_n^2 \rangle = -0.107 \pm 0.006 \pm 0.001_{\text{mod}}$ (fm^2)	$\langle r_n^2 \rangle = -0.104 \pm 0.004 \pm 0.004_{\text{mod}}$ (fm^2)
Rational group	$\langle r_n^2 \rangle = -0.115 \pm 0.006 \pm 0.002_{\text{mod}}$ (fm^2)	$\langle r_n^2 \rangle = -0.115 \pm 0.005 \pm 0.007_{\text{mod}}$ (fm^2)
$\langle r_n^2 \rangle = -0.111 \pm 0.006 \pm 0.002_{\text{mod}} \pm 0.004_{\text{group}}$ (fm^2)		

Table 8. The extracted $\langle r_n^2 \rangle$ from the fits within a low-Q^2 range when the CLAS measurements are included in the data-set. The final result, from the average of the two groups, is given for the fits in the [0 - 0.3 $(\text{GeV}/c)^2$] range, where the model uncertainties are minimized.

3.2.1 Considerations on the CLAS data uncertainties

The CLAS results agree nicely with the MAMI and JLab/Hall-A measurements within their region of overlap at $Q^2 \approx 0.2$ $(\text{GeV}/c)^2$. Nevertheless, we have some concerns that part of the CLAS uncertainties may be underestimated. If that is indeed the case, one could risk to bias the $\langle r_n^2 \rangle$ extraction when these data are included in the analysis. More specifically, the CLAS detector has a large acceptance coverage (4π), which offers some advantage as to the extent of the measured phase space. Nevertheless, this advantage comes at the expense of the detector's resolution, when compared to the high resolution spectrometers that have been utilized for the MAMI and the JLab/Hall-A measurements. In the latter case (i.e. at MAMI and in Hall-A) the extended phase space is still covered by sequential measurements of the high resolution spectrometers so that one can ultimately achieve an extended phase space coverage that is comparable to the CLAS one. Thus one would naturally expect that ultimately the MAMI and the JLab/Hall-A results will offer smaller overall experimental uncertainties in the transition form factor extraction since e.g. the expectation is that the limitations in the resolution of CLAS will introduce a higher level of systematic uncertainties in the CLAS results. But this is not what one observes in the reported results. The CLAS measurements are reported with superior experimental uncertainties (compared to the MAMI and the Hall-A data). This points out to a potential underestimation for part of the experimental uncertainties within the global fit analysis of the extended CLAS 4π acceptance. We here note that the extraction of the quadrupole transition amplitudes involves a very delicate task since it requires to isolate and extract a very small amplitude from multiple interfering background processes of similar magnitude. A second concern involves the level of the model uncertainties of the CLAS data. One would naturally expect that these uncertainties are not very much dependent on the experimental setup, and that they will be approximately at the same

α_1	α_2	b_1	b_2	b_3	b_4
0.13830±0.12960	0.00011±0.00093	11.1944±0.14021	19.6659±1.1020	30.5455± 5.45231	2.35740 ±0.03289

Table 9. The G_E^p fitted parameters.

level for all data sets (CLAS, Hall-A, MAMI) at the same kinematics. Nevertheless, the reported CLAS model uncertainties are nearly a factor of two smaller compared to the ones of MAMI and Hall-A. One can note here that in the CLAS analysis only two theoretical models have been utilized for the quantification of the model uncertainties, while for the MAMI and Hall-A data a more complete study that engages four theoretical models, as well as an experimental measurement of the photon excitation channel has been considered (as described in detail in earlier sections of this document). Taking the above considerations into account one may wonder if the small improvement that we observe in the r_n uncertainty by the inclusion of the CLAS data, in a region where G_E^n data already exist, is affected by a potential underestimation of these uncertainties. In any case, and for the completeness of this work, we report the results for the $\langle r_n^2 \rangle$ extraction when the CLAS data are included in the analysis, but we suggest that this result is considered with a level of reservation.

4 Flavor dependent charge densities

The neutron and the proton charge densities[39] can be extracted in the infinite-momentum-frame through:

$$\rho(b) = \int_0^\infty \frac{dQ\, Q}{2\pi} J_0(Qb) \frac{G_E(Q^2) + \tau G_M(Q^2)}{1+\tau} \tag{7}$$

where b is the transverse distance, $\tau = Q^2/4m^2$ and J_0 a cylindrical Bessel function. From the neutron and proton densities, invoking charge symmetry, and neglecting the $s\bar{s}$ contribution, we then extract the u- and d-quark densities, where

$$\rho_u(b) = \rho_p(b) + \rho_n(b)/2 \qquad \rho_d(b) = \rho_p(b) + 2\rho_n(b). \tag{8}$$

Here we utilize the most recent parametrizations of the nucleon form factors For the neutron electric form factor we use the parametrization determined in this work i.e. the one of Eq. 5 (Table 6). For the two magnetic form factors,G_M^p and G_M^n, we use[16]. For G_E^p we have performed an updated parametrization for the following reasons: i) so that we may include important and recent measurements from[40] that were not yet available in[16], and ii) so that we may eliminate a constraint on the proton charge radius that has been enforced in[16], which is not in agreement with recent measurements of the proton charge radius. Here we have used a form that has been widely adopted in the past:

$$G_E^p = \frac{1 + (\sum_{i=1}^{2} a_i x_i^i)}{(1 + \sum_{j=1}^{4} b_j x_j^j)} \tag{9}$$

The G_E^p fitted parameters that we have derived are given in Table 9.

The extracted neutron (ρ_n), proton (ρ_p) are presented in Fig. 8. The u- and d-quark densities with their experimental uncertainties are shown at Fig. 5c in the paper. The uncertainties in the charge density distributions result from the uncertainties of the nucleon form factor parametrizations, as they have been determined from the fits of the experimental data. Our data, being particularly sensitive to neutrons long distance structure, offer a factor of 2 improvement in the precision of the neutron charge density at its surface as seen in Fig. 9.

5 Lattice QCD results

To extract the lattice data presented in this work, we utilize results from a recent calculation by the Extended Twisted Mass Collaboration (ETMC) on the electromagnetic form factors of the proton and neutron[41]. The lattice calculation is pioneering in more than one ways. First, the numerical simulations have been performed taking into account the up, down, strange and charm quark in the sea ($N_f = 2+1+1$ of twisted mass clover-improved fermions). All of the quark masses are fixed to their physical value (physical point), reproducing a pion mass of 139 MeV. In addition, the ensemble has volume with spatial extent 5.12 fm, and a lattice spacing $a = 0.08$ fm. Having a lattice spacing below 0.1 fm, and a large volume, significantly suppressed finite-volume effects and discretizations effects.

Beyond the state-of-the-art ensemble used for the calculation of the electromagnetic form factors, several sources of systematic uncertainties are controlled. Dominance of ground state is reliably established by three methods to analyze the data:

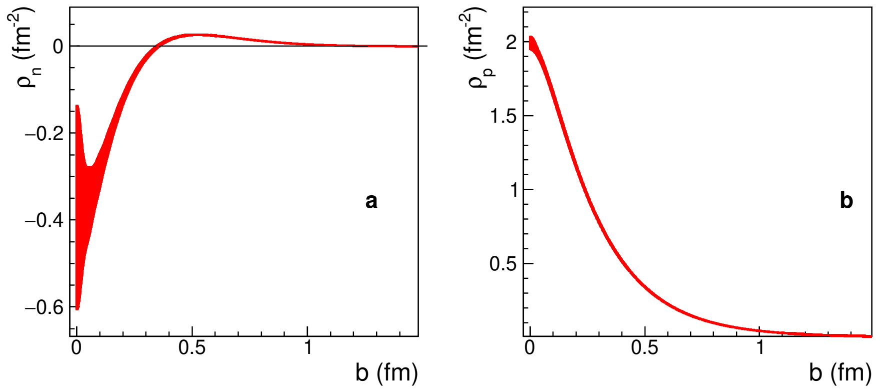

Figure 8. The neutron charge density (panel a) and the proton charge density (panel b).

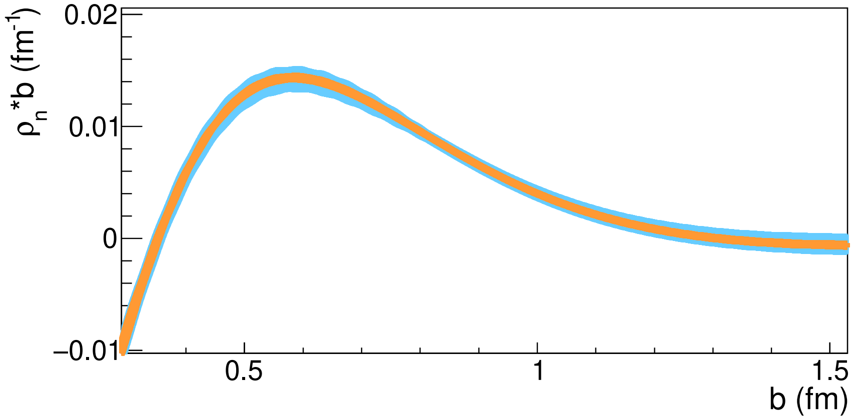

Figure 9. The neutron charge density at it's surface, as derived with (without) our G_E^n data is shown by the inner (outer) band.

single-state fit, two-state fit, and the so-called summation method[42,43]. Furthermore, both the connected and disconnected contributions are included to extract the proton and neutron electromagnetic form factors at the physical point. The achieved accuracy for both connected and disconnected contributions is very high, which is a very challenging task for simulations at the physical point. In fact, it is also shown that the disconnected contributions are non-negligible, reaching up to 15%. Along the same lines, the strange quark contribution to the electromagnetic form factors has been calculated[44], which is, however, beyond the scope of this work.

Here we used the aforementioned data to extract the ratio between the neutron electric and magnetic form factor (G_E^n/G_M^n). The results are shown in Table 10. The very good agreement of the results with the experimental data indicates that lattice QCD has advanced significantly, leading to results of high reliability. The errors include both statistical and systematic uncertainties added in quadrature. For the ratios we perform a jackknife statistical-error analysis, and an error propagation analysis using the individual data on G_E^n and G_M^n. The resulting mean values and errors are almost identical between the two methods.

$Q^2(\mathrm{GeV}/c)^2$	$\frac{G_E^n}{G_M^n}$	$\delta(\frac{G_E^n}{G_M^n})$
0.057	0.0116	0.0032
0.113	0.0198	0.0040
0.167	0.0298	0.0060
0.219	0.0298	0.0077
0.271	0.0427	0.0071
0.321	0.0543	0.0084
0.417	0.0618	0.0134
0.464	0.0688	0.0137
0.510	0.0639	0.0162
0.554	0.0658	0.0176
0.598	0.0797	0.0300
0.642	0.0751	0.0228
0.684	0.0889	0.0251
0.767	0.1167	0.0715
0.807	0.0702	0.0397
0.847	0.0928	0.0403
0.886	0.1392	0.0626
0.925	0.1267	0.0638
0.963	0.0916	0.0571

Table 10. Lattice results for $\frac{G_E^n}{G_M^n}$.

References

1. Blomberg, A. *et al.* Electroexcitation of the $\Delta^+(1232)$ at low momentum transfer. *Phys. Lett.* **B760**, 267–272 (2016).
2. Stave, S. *et al.* Lowest Q^2 Measurement of the $\gamma *$ p —> Δ Reaction: Probing the Pionic Contribution. *Eur. Phys. J.* **A30**, 471–476 (2006).
3. Sparveris, N. *et al.* Measurements of the γ^*p $\rightarrow$ Δ reaction at low Q^2. *Eur. Phys. J.* **A49**, 136 (2013).
4. Sparveris, N. F. *et al.* Determination of quadrupole strengths in the $\gamma * p \rightarrow \Delta(1232)$ transition at $Q^2 = 0.20\ (GeV/c)^2$. *Phys. Lett.* **B651**, 102–107 (2007).
5. Blomberg, A. *et al.* Virtual Compton Scattering measurements in the nucleon resonance region. *Eur. Phys. J. A* **55**, 182 (2019). 1901.08951.
6. Aznauryan, I. G. *et al.* Electroexcitation of nucleon resonances from CLAS data on single pion electroproduction. *Phys. Rev.* **C80**, 055203 (2009).
7. Kamalov, S. & Yang, S. N. Pion cloud and the Q^2 dependence of $\gamma *$ N <—> Δ transition form-factors. *Phys. Rev. Lett.* **83**, 4494–4497 (1999).
8. Sato, T. & Lee, T. Dynamical study of the Delta excitation in N (e, e-prime pi) reactions. *Phys. Rev. C* **63**, 055201 (2001).
9. Drechsel, D., Hanstein, O., Kamalov, S. S. & Tiator, L. A Unitary isobar model for pion photoproduction and electroproduction on the proton up to 1-GeV. *Nucl. Phys.* **A645**, 145–174 (1999).
10. Kamalov, S., Chen, G.-Y., Yang, S.-N., Drechsel, D. & Tiator, L. Pi0 photoproduction and electroproduction at threshold within a dynamical model. *Phys. Lett. B* **522**, 27–36 (2001).
11. Arndt, R. A., Briscoe, W. J., Strakovsky, I. I. & Workman, R. L. Analysis of pion photoproduction data. *Phys. Rev.* **C66**, 055213 (2002).
12. Buchmann, A. J. Electromagnetic N —> Δ transition and neutron form-factors. *Phys. Rev. Lett.* **93**, 212301 (2004).
13. Pascalutsa, V. & Vanderhaeghen, M. Large-N_c relations for the electromagnetic nucleon-to-Δ form factors. *Phys. Rev.* **D76**, 111501(R) (2007).
14. Riordan, S. *et al.* Measurements of the Electric Form Factor of the Neutron up to Q^2=3.4 GeV^2 using the Reaction $^3\vec{He}(\vec{e},e'n)pp$. *Phys. Rev. Lett.* **105**, 262302 (2010).

15. Geis, E. *et al.* The Charge Form Factor of the Neutron at Low Momentum Transfer from the $^2\vec{H}(\vec{e},e'n)p$ Reaction. *Phys. Rev. Lett.* **101**, 042501 (2008).

16. Ye, Z., Arrington, J., Hill, R. J. & Lee, G. Proton and Neutron Electromagnetic Form Factors and Uncertainties. *Phys. Lett.* **B777**, 8–15 (2018).

17. Kelly, J. J. Simple parametrization of nucleon form factors. *Phys. Rev.* **C70**, 068202 (2004).

18. Sparveris, N. F. *et al.* Investigation of the conjectured nucleon deformation at low momentum transfer. *Phys. Rev. Lett.* **94**, 022003 (2005).

19. Elsner, D. *et al.* Measurement of the LT-asymmetry in π^0 electroproduction at the energy of the $\Delta(1232)$ resonance. *Eur. Phys. J.* **A27**, 91–97 (2006).

20. Kelly, J. J. *et al.* Recoil polarization measurements for neutral pion electroproduction at $Q^2 = 1\ (GeV/c)^2$ near the Delta resonance. *Phys. Rev.* **C75**, 025201 (2007).

21. Madey, R. *et al.* Measurements of G_E^n/G_M^n from the $^2H(\vec{e},e'\vec{n})^1H$ reaction to $Q^2 = 1.45\ (GeV/c)^2$. *Phys. Rev. Lett.* **91**, 122002 (2003).

22. Schlimme, B. S. *et al.* Measurement of the neutron electric to magnetic form factor ratio at $Q^2 = 1.58\ GeV^2$ using the reaction $^3\vec{H}e(\vec{e},e'n)pp$. *Phys. Rev. Lett.* **111**, 132504 (2013).

23. Glazier, D. I. *et al.* Measurement of the electric form-factor of the neutron at $Q^2 = 0.3\ (GeV/c)^2$ to $0.8\ (GeV/c)^2$. *Eur. Phys. J.* **A24**, 101–109 (2005).

24. Plaster, B. *et al.* Measurements of the neutron electric to magnetic form-factor ratio G_{En}/G_{Mn} via the $^2H(\vec{e},e',\vec{n})^1H$ reaction to Q^2 =1.45 $(GeV/c)^2$. *Phys. Rev.* **C73**, 025205 (2006).

25. Zhu, H. *et al.* A Measurement of the electric form-factor of the neutron through $\vec{d}(\vec{e},e'n)p$ at $Q^2 = 0.5\ (GeV/c)^2$. *Phys. Rev. Lett.* **87**, 081801 (2001).

26. Warren, G. *et al.* Measurement of the electric form-factor of the neutron at $Q^2 = 0.5$ and $1.0\ GeV^2/c^2$. *Phys. Rev. Lett.* **92**, 042301 (2004).

27. Rohe, D. *et al.* Measurement of the neutron electric form-factor $G_{(en)}$ at $0.67(GeV/c)^2$ via $^3\vec{H}e(\vec{e},e'n)$. *Phys. Rev. Lett.* **83**, 4257–4260 (1999).

28. Bermuth, J. *et al.* The Neutron charge form-factor and target analyzing powers from $^3\vec{H}e(\vec{e},e'n)$ scattering. *Phys. Lett.* **B564**, 199–204 (2003).

29. Eden, T. *et al.* Electric form factor of the neutron from the $^2\vec{H}(\vec{e}$,e′n)^1H reaction at Q^2=0.255 $(GeV/c)^2$. *Phys. Rev. C* **50**, R1749–R1753 (1994).

30. Ostrick, M. *et al.* Measurement of the Neutron Electric Form Factor $G_{E,n}$ in the Quasifree $^2H(\vec{e},e'\vec{n})p$ Reaction. *Phys. Rev. Lett.* **83**, 276–279 (1999).

31. Golak, J., Ziemer, G., Kamada, H., Witała, H. & Glöckle, W. Extraction of electromagnetic neutron form factors through inclusive and exclusive polarized electron scattering on a polarized ^{3}He target. *Phys. Rev. C* **63**, 034006 (2001).

32. Herberg, C. *et al.* Determination of the neutron electric form-factor in the D(e,e' n)p reaction and the influence of nuclear binding. *Eur. Phys. J.* **A5**, 131–135 (1999).

33. Galster, S. *et al.* Elastic electron-deuteron scattering and the electric neutron form factor at four-momentum transfers $5\text{fm}^{-2} < q^2 < 14\text{fm}^{-2}$. *Nucl. Phys. B* **32**, 221–237 (1971).

34. Gentile, T. & Crawford, C. Neutron charge radius and the neutron electric form factor. *Phys. Rev. C* **83**, 055203 (2011).

35. Kopecky, S. *et al.* Neutron charge radius determined from the energy dependence of the neutron transmission of liquid Pb-208 and Bi-209. *Phys. Rev.* **C56**, 2229–2237 (1997).

36. Koester, L. *et al.* Neutron electron scattering length and electric polarizability of the neutron derived from cross-sections of bismuth and of lead and its isotopes. *Phys. Rev.* **C51**, 3363–3371 (1995).

37. Aleksandrov, Yu. A., Vrana, M., Manrique, G. J., Machekhina, T. A. & Sedlakova, L. N. Neutron rms radius and electric polarizability from data on the interaction of slow neutrons with bismuth. *Sov. J. Nucl. Phys.* **44**, 900–902 (1986).

38. Krohn, V. E. & Ringo, G. R. Reconsiderations of the electron - neutron scattering length as measured by the scattering of thermal neutrons by noble gases. *Phys. Rev.* **D8**, 1305–1307 (1973).

39. Miller, G. A. Charge Density of the Neutron and Proton . *Phys. Rev. Lett.* **99**, 112001 (2007).

40. Xiong, W. *et al.* A small proton charge radius from an electron–proton scattering experiment. *Nature* **575**, 147–150 (2019).

41. Alexandrou, C. *et al.* Proton and neutron electromagnetic form factors from lattice QCD. *Phys. Rev.* **D100**, 014509 (2019).

42. Maiani, L., Martinelli, G., Paciello, M. & Taglienti, B. Scalar Densities and Baryon Mass Differences in Lattice QCD With Wilson Fermions. *Nucl. Phys. B* **293**, 420, DOI: 10.1016/0550-3213(87)90078-2 (1987).

43. Capitani, S. *et al.* The nucleon axial charge from lattice QCD with controlled errors. *Phys. Rev. D* **86**, 074502, DOI: 10.1103/PhysRevD.86.074502 (2012). 1205.0180.

44. Alexandrou, C. *et al.* Nucleon strange electromagnetic form factors. *Phys. Rev. D* **101**, 031501, DOI: 10.1103/PhysRevD.101.031501 (2020). 1909.10744.
